# Involvement of Macrophage Inflammatory Protein-1 Family Members in the Development of Diabetic Neuropathy and Their Contribution to Effectiveness of Morphine

**DOI:** 10.3389/fimmu.2018.00494

**Published:** 2018-03-12

**Authors:** Ewelina Rojewska, Magdalena Zychowska, Anna Piotrowska, Grzegorz Kreiner, Irena Nalepa, Joanna Mika

**Affiliations:** ^1^Department of Pain Pharmacology, Institute of Pharmacology, Polish Academy of Sciences, Krakow, Poland; ^2^Department of Brain Biochemistry, Institute of Pharmacology, Polish Academy of Sciences, Krakow, Poland

**Keywords:** neutralizing antibody, CCL3, CCL4, CCL9, CCR1 antagonist (J113863)

## Abstract

Current investigations underline the important roles of C–C motif ligands in the development of neuropathic pain; however, their participation in diabetic neuropathy is still undefined. Therefore, the goal of our study was to evaluate the participation of macrophage inflammatory protein-1 (MIP-1) family members (CCL3, CCL4, CCL9) in a streptozotocin (STZ)-induced mouse model of diabetic neuropathic pain. Single intrathecal administration of each MIP-1 member (10, 100, or 500 ng/5 μl) in naïve mice evoked hypersensitivity to mechanical (von Frey test) and thermal (cold plate test) stimuli. Concomitantly, protein analysis has shown that, 7 days following STZ injection, the levels of CCL3 and CCL9 (but not CCL4) are increased in the lumbar spinal cord. Performed additionally, immunofluorescence staining undoubtedly revealed that CCL3, CCL9, and their receptors (CCR1 and CCR5) are expressed predominantly by neurons. *In vitro* studies provided evidence that the observed expression of CCL3 and CCL9 may be partially of glial origin; however, this observation was only partially possible to confirm by immunohistochemical study. Single intrathecal administration of CCL3 or CCL9 neutralizing antibody (2 and 4 μg/5 μl) delayed neuropathic pain symptoms as measured at day 7 following STZ administration. Single intrathecal injection of a CCR1 antagonist (J113863; 15 and 20 μg/5 μl) also attenuated pain-related behavior as evaluated at day 7 after STZ. Both neutralizing antibodies, as well as the CCR1 antagonist, enhanced the effectiveness of morphine in STZ-induced diabetic neuropathy. These findings highlight the important roles of CCL3 and CCL9 in the pathology of diabetic neuropathic pain and suggest that they play pivotal roles in opioid analgesia.

## Introduction

Diabetes mellitus is the first non-infectious disease recognized as an epidemic of the twenty-first century [United Nations Resolution 61/225]. One of its main consequences is peripheral nerve damage, which leads to the development of neuropathic pain (1). Consequently, ulcers and infections may develop on the feet, sometimes leading to amputation. The mechanisms involved in the development of neuropathy are complex (2); however, the participation of immune factors in this phenomenon is currently receiving a great deal of attention (3–6). It is well established that glial cells, especially microglia, are responsible for its initiation. Additionally, these cells, when activated, release a broad spectrum of nociceptive factors together with cytokines; among those molecules, chemokines seem to be especially important. Recently, it was postulated that C–C (CCL2, CCL3), C–X–C (CXCL1, CXCL5, CXCL12), and X–C (XCL1) motif chemokine ligands play a crucial role in the development of neuropathic pain, including the pain accompanying diabetes (7–10). Therefore, determining the role of C–C motif chemokine ligands in the MIP-1 family (namely, CCL3, CCL4, CCL9) in the development of diabetic neuropathy is highly important. Streptozotocin (STZ)-induced diabetes is a well-established animal model used in studies on neuropathic pain (4, 10, 11). It has already been shown that STZ administration increases blood glucose concentration, which is correlated with the development of long-lasting hypersensitivity to mechanical and thermal stimuli (6, 12, 13).

It was postulated that autoantibodies against CCL3 are biomarkers of type 1 diabetes development (14). CCL3, also known as macrophage inflammatory protein-1-alpha, belongs to the MIP-1 family, which, in the case of rodents, contains two other members: CCL4 (MIP-1-beta) and CCL9 (MIP-1-gamma). The MIP-1 chemokines exert their biological effects through G-protein-coupled receptors (15). It has been shown that CCL3 and CCL4 can participate in the development of neuropathic pain after peripheral nerve injury (7, 16), and CCL9 seems to be important in a model of chronic joint pain (17). These findings underline the important roles of MIP-1 family members in neuropathy; however, their function in neuropathic pain accompanying diabetes needs to be elucidated.

There are existing medications available for the treatment of neuropathic pain symptoms accompanying diabetes; however, most of the patients who take them have an unsatisfactory response or experience adverse reactions. Recent studies have shown that modulating the levels of endogenous pro- and antinociceptive factors by minocycline treatment, for example, results in mitigation of pain-related behavior in a model of diabetic neuropathy (4, 6, 10). Additionally, neutralization of endogenous chemokines, such as CCL1, CXCL1, or XCL1, by their neutralizing antibodies (nAbs) also provides a beneficial analgesic effect (10, 18, 19). Currently, researchers are also focused on antagonizing the chemokine receptors as a possible method of neuropathic pain treatment. It was previously shown that RS504393 (a CCR2 antagonist) and maraviroc (a CCR5 antagonist) both relieve neuropathic pain symptoms in a rat model of neuropathy (8, 20, 21) and simultaneously enhance the effectiveness of morphine (22, 23). Therefore, it is tempting to verify the roles of MIP-1 receptors in the development of diabetic neuropathic pain and the viability of a potential new analgesic strategy based on blocking those receptors.

The aim of our study was to examine whether the intrathecal injection of MIP-1 members may enhance nociceptive transmission in naïve mice. Additionally, we verified on the molecular level whether there were any changes in the protein abundance of MIP-1 members and their most important receptors [selected by their degree of affinity for CCL3 (CCR1 and CCR5), CCL4 (CCR1 and CCR5), or CCL9 (CCR1)] in the lumbar spinal cord of mice with a STZ-induced diabetes. Additionally, we studied the cellular source of MIP-1 ligands in primary glial cell cultures and the presence of CCR1/CCR5 on these cells. Immunohistochemical staining was performed to define the cellular localization of both ligands and receptors in the lumbar spinal cord of mice with STZ-induced diabetes. Furthermore, by administration of CCL3 or CCL9 neutralizing antibody, we traced the influence of those chemokines on neuropathic pain symptoms developed in STZ-injected mice and studied whether this neutralization can enhance the analgesic properties of morphine. We also investigated whether J113863 (a CCR1 antagonist) and DAPTA (CCR5 antagonist) have any potential in relieving neuropathic pain symptoms developed after STZ administration and enhancing the effectiveness of morphine.

## Materials and Methods

### Animals

All experiments were performed on male Albino-Swiss mice (20–22 g; age: 32–38 days) purchased from Charles River (Germany). The animals were housed in cages with sawdust under a 12/12 h light/dark cycle. Food and water were available *ad libitum*. The experiments were carried out according to IASP recommendations (24) and the NIH Guide for the Care and Use of Laboratory Animals and were approved by the second Local Ethical Committee on Animal Testing at the Institute of Pharmacology, Polish Academy of Sciences (LKE 1277/2015 and 75/2017).

### Mouse Model of Diabetic Neuropathic Pain

STZ is one of the most prominent diabetogenic chemical components in experimental diabetes research (25–33). Single intraperitoneal (*i.p*.) administration of STZ (200 mg/kg; Sigma-Aldrich, USA), dissolved in water for injections, was used to generate a type 1 diabetes model (6, 9, 10, 31, 34). The STZ evoked diabetic neuropathy by the specific necrosis of the pancreatic beta cells (34, 35). Additionally, age-matched non-diabetic (naïve) mice received water for injections. Blood was collected from the tail veins of the mice, and the glucose concentration was measured with an Accu-Chek Active glucometer. High levels of plasma glucose were observed as early as one day after the injection of STZ. Mice were considered diabetic when serum glucose levels were higher than 300 mg/dl at day 7 after STZ injection. The behavioral tests were performed on day 7 following STZ administration.

### Behavioral Tests

#### Mechanical Threshold—*von Frey Test*

Calibrated nylon monofilaments (Stoelting, USA) were used to measure the reactions of the mice to mechanical stimuli. Mice were placed in a plastic cage with a wire mesh floor and adapted to the conditions of the experiment for 15 min. Von Frey filaments of increasing strength (from 0.6 to 6 g) were applied sequentially to the plantar surface of the hind paws of each mouse. The measurement was conducted until the hind paw was withdrawn (6, 9, 36).

#### Thermal Threshold—*Cold Plate Test*

A cold plate test (Cold/Hot Plate Analgesia Meter, Columbus Instruments, USA) was used to assess the reactions of the mice to thermal stimuli (6, 9, 37). The mice were put on a cold plate with a temperature of 2°C. The latency to hind paw elevation was noted. The cutoff latency was 30 s.

### Pharmacological Study

#### Intrathecal Administration

Intrathecal (*i.t*.) administration is a standard procedure in our laboratory (9, 10) and is performed using a Hamilton syringe with a thin needle as previously described (38). Substances that were used in the experiments were injected in the lumbar portion of the spinal cord (between the L5 and L6 vertebrae) in a volume of 5 µl, and the tail reflex was an indicator of correct administration.

##### Single *i.t*. Administration of CCL3, CCL4, or CCL9 in Naïve Mice

CCL3 and CCL9 were obtained from Sigma-Aldrich (USA), and CCL4 was attained from R&D Systems (USA). All chemokines were dissolved in water for injection. After reconstitution, chemokines were singly administered *i.t*. to naïve mice in the following doses: 10, 100, and 500 ng/5 μl. The behavioral tests were performed 60, 240, and 1,440 min following injection.

##### Single *i.t*. Administration of CCL3 or CCL9 Neutralizing Antibody in Mice With STZ-Induced Diabetic Neuropathy

Anti-mouse CCL3 and CCL9 nAbs were obtained from R&D Systems and reconstituted in water for injections. After reconstitution, the nAbs were singly administered *i.t*. to mice on day 7 after STZ injection in the following doses: 0.5, 2, and 4 μg/5 μl. Additionally, the control group was injected with 5 µl of water for injection per mouse. The behavioral tests were performed 60, 240, 1,440, and 2,880 min following injection of nAbs. Additionally, at day 7 following STZ administration, for the doses of 2 μg/5 μl nAb CCL3 and 4 μg/5 μl nAb CCL9, the same behavioral tests were also performed after 120 min following antibody administration.

##### Single *i.t*. CCR1 (J113863) or CCR5 (DAPTA) Antagonist Administration in Mice With STZ-Induced Diabetic Neuropathy

J113863, a potent CCR1 antagonist, was obtained from TOCRIS (UK) and dissolved in 5% dimethyl sulfoxide (DMSO). DAPTA (d-Ala–Ser–Thr–Thr–Thr–Asn–Tyr–Thr-NH_2_), a selective CCR5 antagonist, was obtained from TOCRIS (UK) and dissolved in water for injection. After reconstitution, the antagonists were singly administered *i.t*. to mice on day 7 after STZ injection in the following doses: 10, 15, and 20 μg/5 μl in the case of J113863 and 10 and 30 μg/5 μl in the case of DAPTA. The control groups received 5 µl of 5% DMSO or 5 µl of water for injection, respectively. The behavioral tests were performed after 30 min following antagonist injection. Additionally, for J113863 (dose 15 μg/5 μl) and DAPTA (dose 30 μg/5 μl), the same behavioral tests were also performed after 90, 240, and 1,440 min following antagonist administration.

##### Single *i.t*. Morphine Administration in Mice With STZ-Induced Diabetic Neuropathy

Morphine was obtained from Sigma-Aldrich (USA) and dissolved in water for injection at a final concentration of 1 μg/5 μl. Morphine was singly administered *i.t*. 135 min after injection of CCL3 or CCL9 neutralizing antibody (2 μg/5 μl and 4 μg/5 μl, respectively) on day 7 after STZ administration. The control group was injected with 5 µl of water for injection per mouse. The behavioral tests were first performed 120 min following neutralizing antibody injection and then repeated 30 min after morphine administration (scheme in Figure 7A). Additionally, at day 7 following STZ injection, a single *i.t*. administration of morphine was performed 35 min after J113863 injection (15 μg/5 μl). The control group was injected with 5 µl of 5% DMSO or water for injection per mouse. The behavioral tests were first performed 30 min following J113863 injection and then repeated 30 min after morphine administration (Scheme in Figure 8E).

### Primary Microglial and Astroglial Cultures

Primary cultures of microglia and astroglia were used in our *in vitro* studies. Both types of cell culture were prepared from Wistar rat pups (1-day old) as previously described (39). The cells were isolated from the cerebral cortex and plated at a density of 3 × 10^5^ cells/cm^2^ in a culture medium composed of DMEM/GlutaMAX/high glucose (Gibco, USA) supplemented with 10% heat-inactivated fetal bovine serum, 0.1 mg/ml streptomycin and 100 U/ml penicillin (Gibco). The cultures were maintained in poly-l-lysine-coated 75-cm^2^ culture flasks at 37°C and 5% CO_2_. After 4 days, the culture medium was changed. The next step involved the recovery of the loosely adherent microglial cells by gentle shaking and centrifugation at 37°C for 24 h (200 rpm) on day 9 and after replacing the medium on day 12. The medium was removed, and the astrocytes were replated in culture dishes, where they were maintained for 3 days and then trypsinized (0.005% trypsin-EDTA solution, Sigma-Aldrich). The microglia/astroglia were resuspended in culture medium, plated at final densities of 2 × 10^5^ cells on 24-well plates for mRNA analysis and 1.2 × 10^6^ cells on 6-well plates for protein analysis, and incubated for 48 h. The primary microglia and astrocyte cultures were stimulated for 24 h using lipopolysaccharide (LPS; 100 ng/ml; Sigma-Aldrich), because it is known from our previous studies that such *in vitro* stimulation correlates well with the changes observed *in vivo* in neuropathic pain models (8, 10, 21–23, 40, 41). To identify the microglia and astrocytes in the *in vitro* cell cultures, we utilized IBA1 (ionized calcium-binding adapter molecule 1) as a microglial marker (anti-IBA1, 1:500, Santa Cruz) and GFAP (glial fibrillary acidic protein) as an astrocyte marker (anti-GFAP, 1:500, Santa Cruz, CA, USA). As a result, we obtained highly homogeneous microglial and astroglial populations that were more than 95% positive for IBA1 and GFAP, respectively. The homogeneities of our cultures were similar to those reported by Zawadzka and Kaminska (39).

### Molecular and Immunohistochemical Analysis

#### Quantitative Reverse Transcriptase Real-time PCR (qRT-PCR)

The primary glial cultures were stimulated for 24 h by the administration of LPS (100 ng/ml) for mRNA analysis. Total RNA was extracted with TRIzol Reagent (Invitrogen, USA) as previously described (42). The RNA concentrations were measured using a NanoDrop ND-1000 Spectrometer (NanoDrop Technologies, USA). Reverse transcription was performed on 1 µg of total RNA from the cultured cells using Omniscript reverse transcriptase (Qiagen Inc., USA) at 37°C for 60 min. The real-time reactions were performed in the presence of an RNAse inhibitor (Promega, USA) and oligo (dT)_16_ primers (Qiagen, Inc.). The cDNA was diluted 1:10 with H_2_O, and for each reaction, ~50 ng of cDNA synthesized from the total RNA template was obtained from each individual animal and used for the qRT-PCR reactions. qRT-PCR was performed using Assay-On-Demand TaqMan probes (Applied Biosystems, USA) and run on an iCycler device (Bio-Rad, Hercules, CA, USA). The amplification efficiency for each assay was determined by running a standard dilution curve. The following TaqMan primers were used: Rn01527840_m1 (*Hprt)*, Rn01464736_g1 (*Ccl3*), Rn00671924_m1 (*Ccl4*), Rn01471276_m1 (*Ccl9*), Rn00571950_s1 (*Ccr1*), and Rn02132969_s1 (*Ccr5*). The expression levels of Hprt were measured in the non-stimulated and LPS-stimulated cells and quantified relative to the control (vehicle) to account for variations in the amounts of cDNA. The Hprt levels did not significantly differ across all groups, and Hprt was, therefore, used as a housekeeping gene control (data not shown). The cycle threshold values were calculated automatically with the software CFX Manager v2.1 using the default parameters. The RNA abundance was calculated as 2^−(threshold cycle)^.

#### RayBio Mouse Inflammation Antibody Array

The lumbar spinal cord (L4-L6) from naïve and STZ-induced diabetic neuropathic mice was removed 7 days after STZ administration. The sample preparation began with homogenization of the tissue [1×Cell Lysis Buffer (RayBio, USA) with Protease Inhibitor Cocktail (Sigma)] and centrifugation (14,000 × *g* for 30 min at 4°C). Then, the protein concentration was evaluated with a BCA Protein Assay Kit (Sigma), and each sample was diluted (1× Blocking Buffer) to a final concentration of 250 µg. The inflammation antibody array membranes (Table 1) were blocked (1 h, room temperature) and then incubated with 1 ml of sample (overnight, 4°C). After incubation and decantation of the samples, the membranes were washed first with 2 ml of 1×Wash Buffer I (RayBio) and then with 2 ml of 1×Wash Buffer II (RayBio). Each membrane was covered with 1 ml of diluted Biotin-Conjugated Anti-Cytokine antibodies (RayBio) and incubated for 90 min (room temperature). After decantation of the primary antibodies, the membranes were washed and incubated (2 h, room temperature) with 2 ml of 1,000-fold diluted HRP-conjugated streptavidin (RayBio). After decantation of the HRP-conjugated streptavidin, the membranes were washed. Immunocomplexes were detected using Detection Buffer (RayBio) and visualized using a Fujifilm LAS 4000 fluorescent imaging system. The relative levels of immunoreactivity were quantified using Fujifilm Image Gage software (6, 9).

**Table 1:** The RayBio^®^ mouse inflammation antibody array 1 (40) was used.

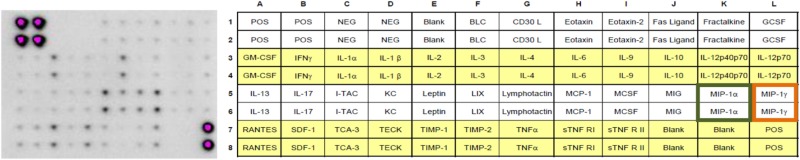

*CCL3 and CCL9 are marked as MIP-1α and MIP-1γ, respectively*.

#### Western Blot

In the naïve and STZ-induced diabetic neuropathic mice, the lumbar (L4-L6) parts of the spinal cords were dissected on day 7 following STZ administration. The cell lysates (in RIPA buffer with a protease inhibitor cocktail) from primary microglial and astroglial cultures for western blot analysis were collected 24 h after LPS stimulation. The samples were homogenized [RIPA buffer with Protease Inhibitor Cocktail (Sigma)] and centrifuged (14,000 × *g* for 30 min), and the protein concentration was determined [BCA Protein Assay Kit (Sigma)]. The samples were diluted to a concentration of 30 µg, heated in Laemmli loading buffer (Bio-Rad, USA) for 8 min at 99°C and resolved by SDS-PAGE on 4–15% polyacrylamide Criterion TGX gels (Bio-Rad). The proteins were transferred to Immun-Blot PVDF membranes (Bio-Rad) and blocked for 60 min in 5% blocking buffer [non-fat dry milk with TBST (Tris-buffered saline with 0.1% TWEEN 20)]. The membranes were incubated for 24 h at 4°C with antibodies diluted with a SignalBoost Immunoreaction Enhancer Kit (Merck Millipore, USA) as follows: rabbit polyclonal anti-IBA1 (1:500; Proteintech, USA), rabbit polyclonal anti-GFAP (1:10,000; NOVUS, USA), rabbit polyclonal anti-CCL3 (1:500; Thermo Fisher Scientific, USA), rabbit polyclonal anti-CCL4 (1:500; ProSci Inc., USA) for mice tissue, rabbit polyclonal nati-CCL4 (1:500; Thermo Fisher Scientific) for primary cell cultures; rabbit polyclonal anti-CCL9 (1:500; Bioss, USA), rabbit polyclonal anti-CCR1 (1:500; NOVUS), rabbit polyclonal anti-CCR5 (1:500; NOVUS), and mouse monoclonal anti-GAPDH (1:5,000; Millipore) as a loading control. After being washed in TBST, the blots were incubated for 1 h at room temperature with HRP-conjugated secondary antibodies diluted at 1:5,000 with a SignalBoost Immunoreaction Enhancer Kit. The immunocomplexes were detected with Clarity Western ECL Substrate (Bio-Rad) and visualized using a Fujifilm LAS 4000 luminescent image analyzer system. The relative levels of immunoreactivity were quantified using Fujifilm Image Gage software (10, 21).

### Immunofluorescence Staining

Immunohistochemistry assays were performed on paraffin-embedded 7-µM-thick microtome sections of lumbar (L4–L6) spinal cords that were removed from diabetic mice on day 7 after STZ administration. Section preparation and immunofluorescence staining were performed as described by Chmielarz et al. (43). Briefly, after deparaffinization and subsequent antigen retrieval (microwave method with citrate buffer), sections from STZ-treated mice were incubated for 30 min in 5% normal pig serum (Vector Labs) in PBST buffer (0.2% Triton X-100 in PBS). Sections were incubated overnight at 4°C with the following primary antibodies: anti-CCL3 (1:50, ThermoFisher Scientific), anti-CCL9 (1:50, Bioss), anti-CCR1 (1:50, Novus), anti-CCR5 (1:50, Novus), anti-NeuN (1:200, Millipore), anti-Iba1 (1:50, Abcam, UK), and anti-GFAP (1:500, Millipore). Antigen-bound primary antibodies were visualized with anti-rabbit Alexa-488, anti-mouse Alexa-594, or anti-chicken Alexa-594 (1:100; Invitrogen) secondary antibodies. Stained sections were examined and photographed under a fluorescence microscope (Nikon Eclipse 50i). The dorsal part of the lumbar spinal cord was visualized in representative images.

### Statistical Analysis

#### The Behavioral Data

**Figure 1:**
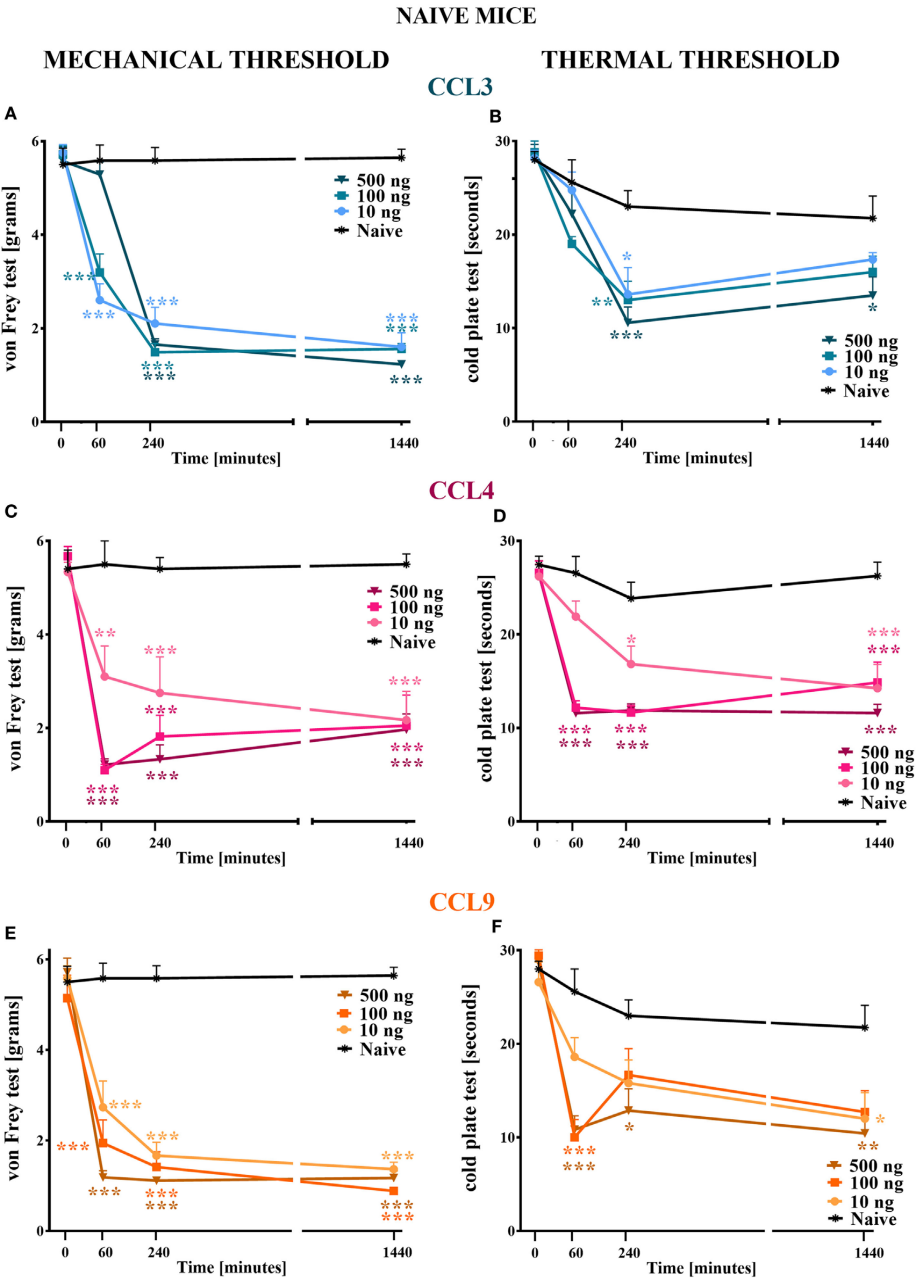
The effects of single intrathecal (*i.t*.) administration of macrophage inflammatory protein-1 family members on nociceptive transmission in naïve mice. The effects of single *i.t*. CCL3 **(A,B)**, CCL4 **(C,D)**, or CCL9 **(E,F)** administration (10, 100, or 500 ng/5 μl) on mechanical nociceptive threshold [von Frey test; **(A,C,E)**] and thermal nociceptive threshold [cold plate test; **(B,D,F)**] were measured at 60, 240, and 1,440 min following administration. The data are presented as the means ± the SEM (4–7 mice per group). The results were evaluated using two-way analysis of variance followed by Bonferroni’s test for multiple comparisons; *(*P* < 0.05), **(*P* < 0.01), and ***(*P* < 0.001) indicate significant differences compared with the naïve animals.

The behavioral data are presented as the means ± SEM. The results were evaluated using two-way analysis of variance (ANOVA) followed by Bonferroni’s test for multiple comparisons (Figures 1 and 7) or one-way ANOVA followed by Bonferroni’s test for multiple comparisons (Figures 8–10). *(*P* < 0.05), **(*P* < 0.01), and ***(*P* < 0.001) indicate significant differences compared with the naïve animals. ^#^(*P* < 0.05), ^##^(*P* < 0.01), and ^###^(*P* < 0.001) indicate significant differences compared with the vehicle (water for injection or 5% DMSO)-injected STZ-induced diabetic neuropathic mice. ^$^(*P* < 0.05), ^$$^(*P* < 0.01), and ^$$$^(*P* < 0.001) indicate significant differences compared with the vehicle (water for injection) + vehicle-treated STZ-induced diabetic neuropathic mice. ^○○^(*P* < 0.01) and ^○○○^(*P* < 0.001) indicate significant differences compared with the neutralizing antibody + vehicle-treated STZ-induced diabetic neuropathic mice. ^&&&^(*P* < 0.001) indicates significant differences compared with the vehicle (water for injection or 5% DMSO) + morphine-treated STZ-induced diabetic neuropathic mice. ^(*P* < 0.05) and ^^^(*P* < 0.001) indicate significant differences compared with the J113863-injected STZ-induced diabetic neuropathic mice.

#### The Molecular Data

**Figure 2:**
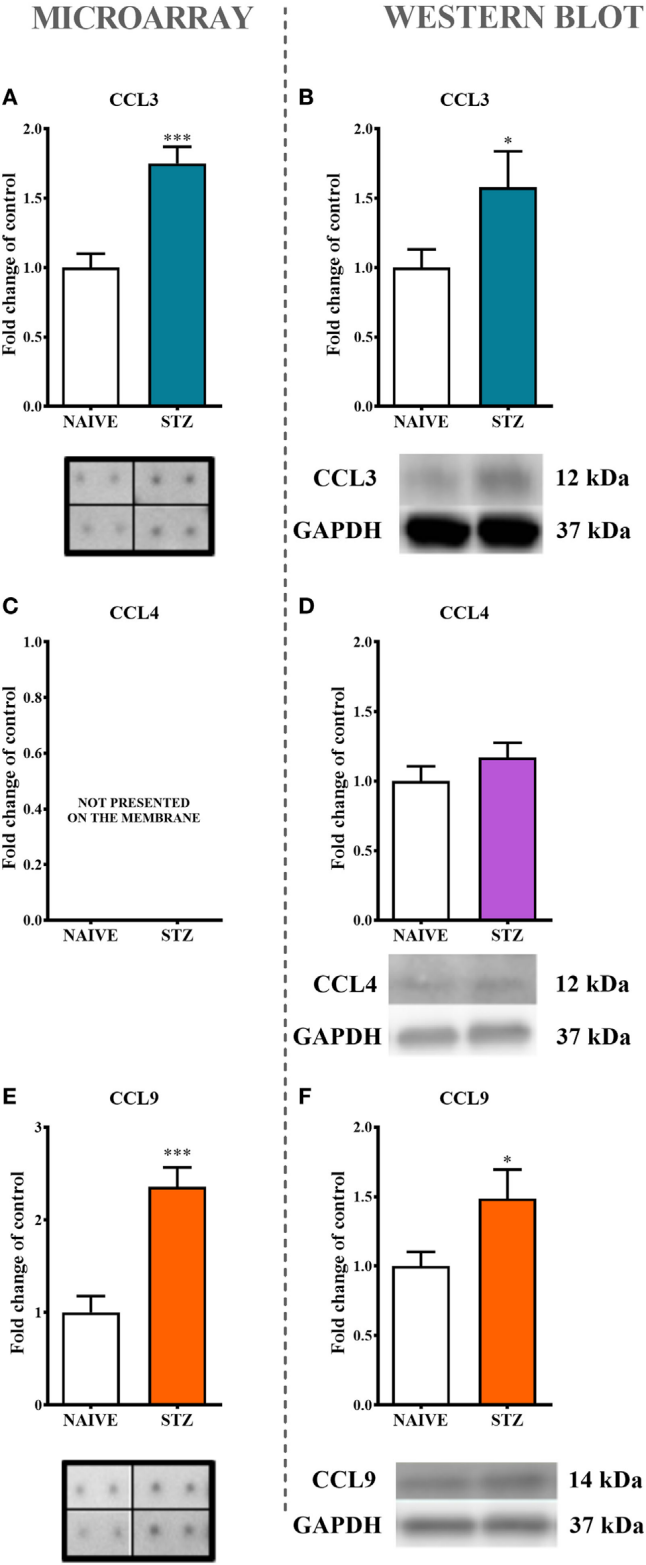
The effects of single intraperitoneal (*i.p*.) streptozotocin (STZ; 200 mg/kg) administration on the protein levels of macrophage inflammatory protein-1 members measured at day 7 following STZ administration in STZ-induced diabetic neuropathic mice. The protein analysis was performed with a protein microarray for CCL3 **(A)** and CCL9 **(E)** and by western blotting for CCL3 **(B)**, CCL4 **(D)**, and CCL9 **(F)**, CCL4 **(C)** not presented on the membrane. Biochemical analysis was performed on lumbar spinal cords dissected from naïve and STZ-injected diabetic neuropathic mice on day 7 following STZ injection. The data are presented as fold changes relative to the control (naïve) ± the SEM (8–12 samples per group). The results were evaluated using Student’s *t*-test; *(*P* < 0.05) and ***(*P* < 0.001) indicate significant differences compared with the control group (naïve animals).

The molecular data are presented as fold changes relative to the controls (naïve/vehicle-treated non-stimulated cells) ± the SEM. The results were evaluated using Student’s *t*-test (Figures 2, 4A,B, 5 and 6). *(*P* < 0.05), **(*P* < 0.01), and ***(*P* < 0.001) indicate significant differences compared with the controls (naïve/vehicle-treated non-stimulated cells). It should be emphasized that all the results of protein expression analysis were not only obtained by two different methodologies (protein arrays and western blot) on newly prepared experimental model (newly isolated tissue), but in each methodological approach, according to the specific requirements of assay, different antibodies had to be used for protein detection. Overall, it were fully independent sets of experiments providing the same feedback.

All graphs were prepared using GraphPad Prism software (version 5.0).

## Results

### The Effect of Single Intrathecal *(i.t.)* Administration of MIP-1 Family Members on Nociceptive Transmission in Naïve Mice

Single *i.t*. administration of MIP-1 family members in each tested dose (10, 100, and 500 ng/5 μl) evoked development of hypersensitivity to mechanical and thermal stimuli as measured by von Frey (Figures 1A,C,E) and cold plate (Figures 1B,D,F) tests, respectively.

#### The Effect of MIP-1 Members on Mechanical Nociceptive Threshold Measured by the von Frey Test

Single *i.t*. administration of CCL3 diminished the nociceptive threshold to mechanical stimuli, and this effect was still observed at 1,440 min following injection (Figure 1A); however, at 60 min following administration of 500 ng CCL3, its pronociceptive effect had not yet been detected (Figure 1A). Additionally, single *i.t*. administration of each CCL4 concentration resulted in the development of hypersensitivity to mechanical stimuli observed up to 1,440 min following administration (Figure 1C). Additionally, after 60 min, two higher doses induced a similar reaction to mechanical stimuli, whereas after 240 min their influence on the nociceptive threshold was dose dependent. After 1,440 min, each dose caused a similar behavioral effect (Figure 1C). Single *i.t*. CCL9 administration in each tested dose evoked development of long-listing hypersensitivity to mechanical stimuli (Figure 1E). At 60 min after CCL9 injection, the reaction to mechanical stimuli varied across concentrations in a dose-dependent manner. Furthermore, this effect was still detected after 1,440 min (Figure 1E).

#### The Effect of MIP-1 Members on Thermal Nociceptive Threshold Measured by the Cold Plate Test

Single *i.t*. administration CCL3 at each concentration lowered the pain perception threshold to thermal stimuli in a dose-dependent manner, and this effect was detected 240 min following injection (Figure 1B). A pronociceptive effect at 1,440 min was only observed for the highest CCL3 concentration (Figure 1B). Additionally, single *i.t*. CCL4 administration produced hypersensitivity to thermal stimuli, and this effect was observed for all tested doses at each time point (Figure 1D). Additionally, the two highest doses (100 and 500 ng) had their strongest and similar effects on thermal nociception at 60 and 240 min following administration. The pronociceptive effects were observed for up to 1,440 min for each CCL4 concentration (Figure 1D). The reaction to thermal stimuli after single *i.t*. CCL9 administration was similar when observed 60 min following injection for the two highest doses (100 and 500 ng); however, after 240 min, the increased pain perception was only detected for the 500 ng dose (Figure 1F). This effect was prolonged up to 1,440 min. Additionally, after 1,440 min, the 10 ng dose also induced development of hypersensitivity to thermal stimuli (Figure 1F).

### The Influence of Single Intraperitoneal (*i.p*.) STZ Administration on MIP-1 Family Member Expression Measured on Day 7 in an STZ-Induced Mouse Model of Diabetic Neuropathic Pain

Single *i.p*. administration of STZ (200 mg/kg) led to increase in blood glucose concentration (535 ± 19mg/dl vs. 166 ± 5 mg/dl in naïve mice) and, in parallel, lowered the pain perception thresholds to mechanical (1.1 ± 0.1g vs. 5.9 ± 0.1g in naïve mice) and thermal (10.2 ± 0.4s vs. 29 ± 0.5s in naïve mice) stimuli as measured by von Frey and cold plate tests, respectively, at day 7 following STZ injection. Western blot analysis, however, indicated that the level of IBA1 was increased (52%) while the GFAP level remained unchanged in STZ-injected mice in comparison to the control group (data not shown on the graphs).

The analysis of protein expression by both a protein array (Figures 2A,E) and western blotting (Figures 2B,F) clearly indicated significant increases in CCL3 (75%, Figure 2A; 57% Figure 2B) and CCL9 (136%, Figure 2E; 49%. Figure 2F) levels in lumbar spinal cords dissected at day 7 after STZ administration. This effect was not observed regarding CCL4 protein (Figure 2D).

### The Spinal Localization of MIP-1 Family Members Determined at Day 7 Following Single Intraperitoneal (*i.p*.) STZ Administration in an STZ-Induced Mouse Model of Diabetic Neuropathic Pain

**Figure 3:**
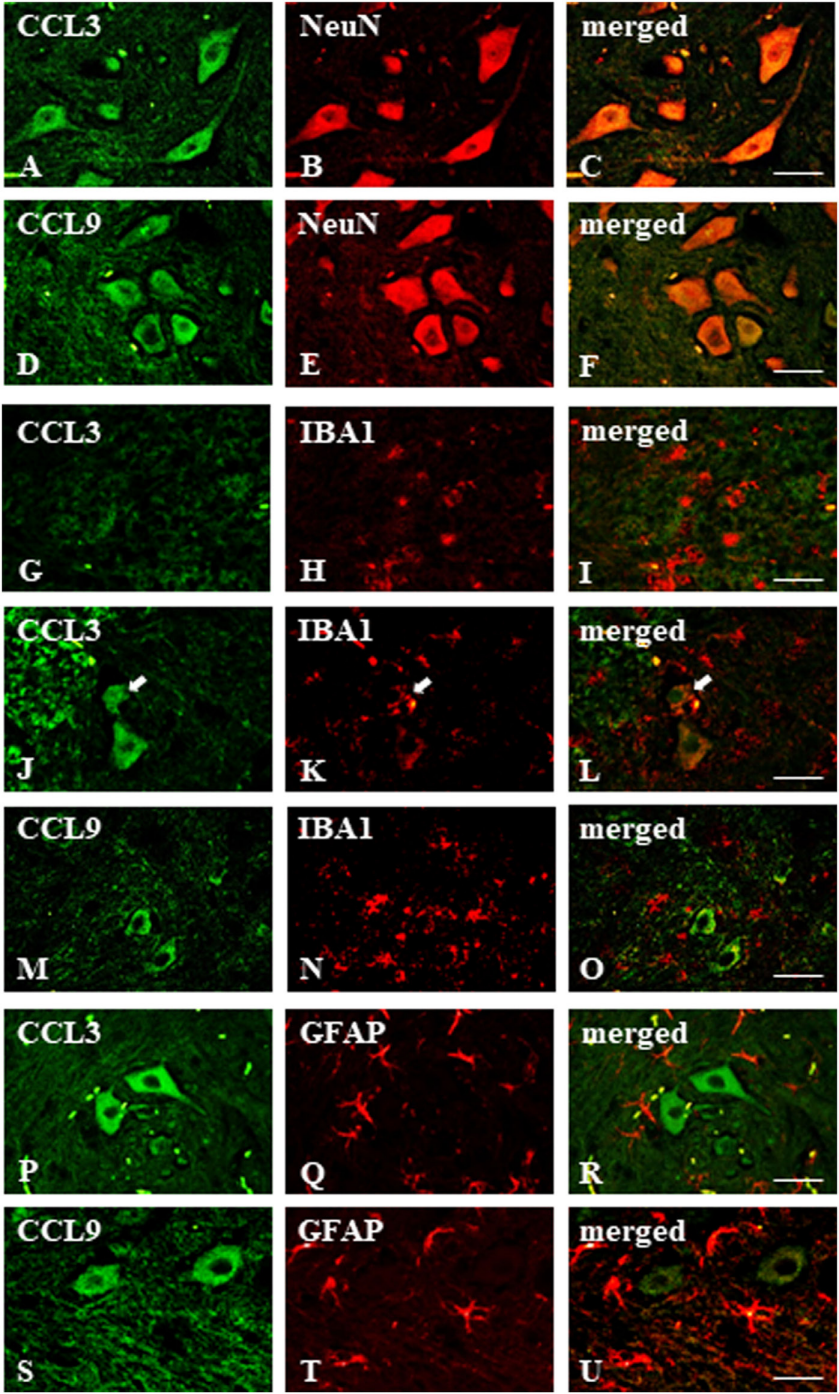
Immunohistochemical staining of macrophage inflammatory protein-1 members in the streptozotocin (STZ)-induced model of diabetic neuropathic pain. Immunofluorescent staining performed on paraffin-embedded microtome slices of spinal cords from STZ-treated mice. **(A–C)**—co-localization of immunoreactivity for CCL3 (green) and NeuN (red); **(D–F)**—co-localization of immunoreactivity for CCL9 (green) and neuronal marker, NeuN (red); **(G–O)**—co-localization of immunoreactivity for CCL3/CCL9 (green) and microglia marker, IBA1 (red); **(P–U)**—co-localization of immunoreactivity for CCL3/CCL9 (green) and astroglia marker, GFAP (red). Scale bars: 25 µm.

The immunofluorescent staining provided evidence that both CCL3 and CCL9 were co-localized with neurons as revealed by co-staining with the neuronal marker NeuN (Figures 3A–F). Their microglial origin, determined previously by *in vitro* study, could not be clearly confirmed; however, apart from IBA1-positive (microglia marker) cells not expressing CCL3 nor CCL9 (Figures 3G–I,M–O, respectively), there were some cells showing coexpression of CCL3 and IBA1 (Figures 3J–L). No co-localization of CCL3 nor CCL9 with the astroglial marker GFAP was observed (Figures 3P–U, respectively). Regarding CCL4, it was not possible to determine its expression due to completely non-specific signal from the antibody used for the immunohistochemical studies performed on paraffin sections (data not shown).

### The Spinal Expression of MIP-1 Family Member Receptors (CCR1 and CCR5) Measured at Day 7 Following Single Intraperitoneal (*i.p*.) STZ Administration in an STZ-Induced Mouse Model of Diabetic Neuropathic Pain

**Figure 4:**
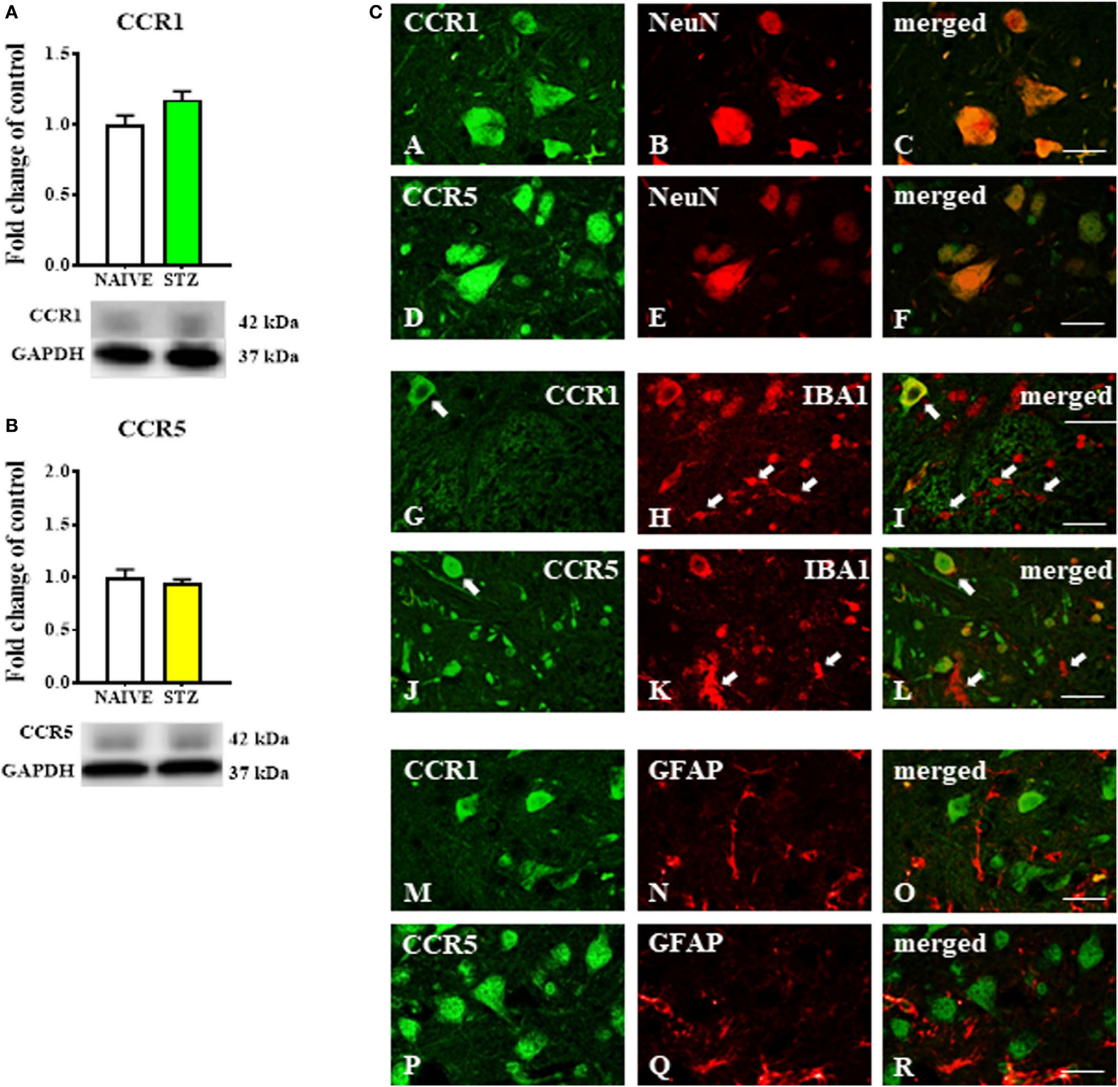
The effects of single intraperitoneal (*i.p*.) streptozotocin (STZ; 200 mg/kg) administration on the protein levels and neuronal localization of CCR1 and CCR5 measured at day 7 following STZ administration in STZ-induced diabetic neuropathic mice. **(A,B)** Protein analysis performed by western blotting for CCR1 **(A)** and CCR5 **(B)**. **(C)** The co-localization of CCR1 and CCR5 with NeuN **(A–F)**, IBA1 **(G–L)**, and GFAP **(M–R)** assessed by immunohistochemical staining and visualized in the superimposed pictures. The data are presented as fold changes relative to the control (naïve) ± the SEM (8–10 samples per group). The results were evaluated using Student’s *t*-test. Scale bars: 25 µm.

The Western blot protein analysis indicated that the levels of CCR1 (Figure 4A) and CCR5 (Figure 4B) were unchanged in spinal cord tissue derived from STZ-injected mice. Immunofluorescent staining revealed that both CCR1 and CCR5 were co-localized with the neuronal marker NeuN (Figures 4A–F, respectively) as visualized on sections taken from lumbar spinal cord (L4-L5) tissue dissected at day 7 after STZ administration. There was no clear co-localization of CCR1 and CCR5 found with either microglial (Figures 4G–L) or astroglial cells (Figures 4M–R) visualized by IBA1 and GFAP markers, respectively.

### The Influences of LPS Stimulation on the mRNA and Protein Levels of MIP-1 Family Members

#### Primary Microglial Cultures

**Figure 5:**
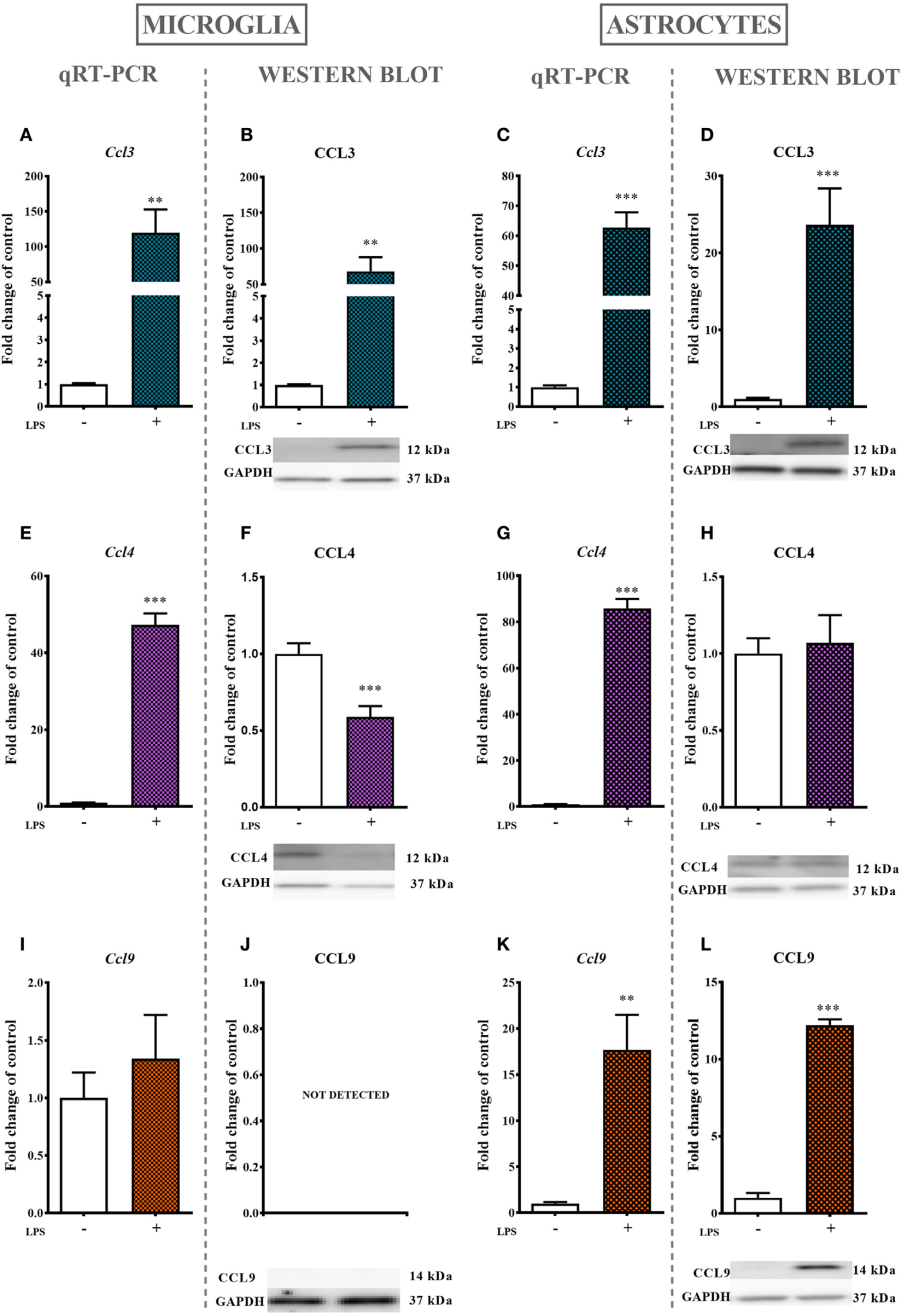
The effects of lipopolysaccharide (LPS; 100 ng/ml) stimulation on the mRNA and protein levels of macrophage inflammatory protein-1 members in primary microglial and astroglial cultures. The mRNA expression of *Ccl3* **(A,C)**, *Ccl4* **(E,G)**, and *Ccl9* **(I,K)** was measured by quantitative reverse transcriptase real-time PCR (qRT-PCR) in vehicle(−)- and LPS(+)-treated microglia **(A,E,I)** and astroglia **(C,G,K)** at 24 h following LPS stimulation. The protein levels of CCL3 **(B,D)**, CCL4 **(F,H)**, and CCL9 **(J,L)** were evaluated by western blotting in vehicle(−)- and LPS(+)-treated microglia **(B,F,J)** and astroglia **(D,H,L)** at 24 h following LPS stimulation. The data are presented as the means ± the SEM (4–12 samples per group). The results were evaluated using Student’s *t*-test; **(*P* < 0.01) and ***(*P* < 0.001) indicate significant differences compared with the vehicle(−)-treated cells.

Lipopolysaccharide (100 ng/ml) stimulation for 24 ho profoundly increased the mRNA expression of *Ccl3* (11,900%, Figure 5A) and *Ccl4* (4,700%, Figure 5E) but had no influence on *Ccl9* (Figure 5I) mRNA expression as evaluated by qRT-PCR. Additionally, the protein level of CCL3 was profoundly increased (6,800%, Figure 5B) and the protein level of CCL4 was decreased (40%, Figure 5F) after 24 h of LPS (100 ng/ml) stimulation as revealed by western blot analysis. No CCL9 protein expression was detected (Figure 5J).

#### Primary Astroglial Cultures

The stimulation of astroglial cultures by LPS (100 ng/ml) for 24 h resulted in a dramatic increase in *Ccl3* (6,200%, Figure 5C), *Ccl4* (8,600%, Figure 5G), and *Ccl9* (1,700%, Figure 4K) mRNA expression as determined by qRT-PCR. Western blot protein analysis further confirmed the observed multiple-fold increase in CCL3 (2,300%, Figure 5D), while the expression of CCL4 remained intact (Figure 5H). In contrast to what was observed in microglial cell cultures, the CCL9 protein level was detected and even increased in astroglial cultures (1,200%, Figure 5L).

### The Influences of LPS Stimulation on the mRNA and Protein Levels of CCR1 and CCR5

#### Primary Microglial Cultures

**Figure 6:**
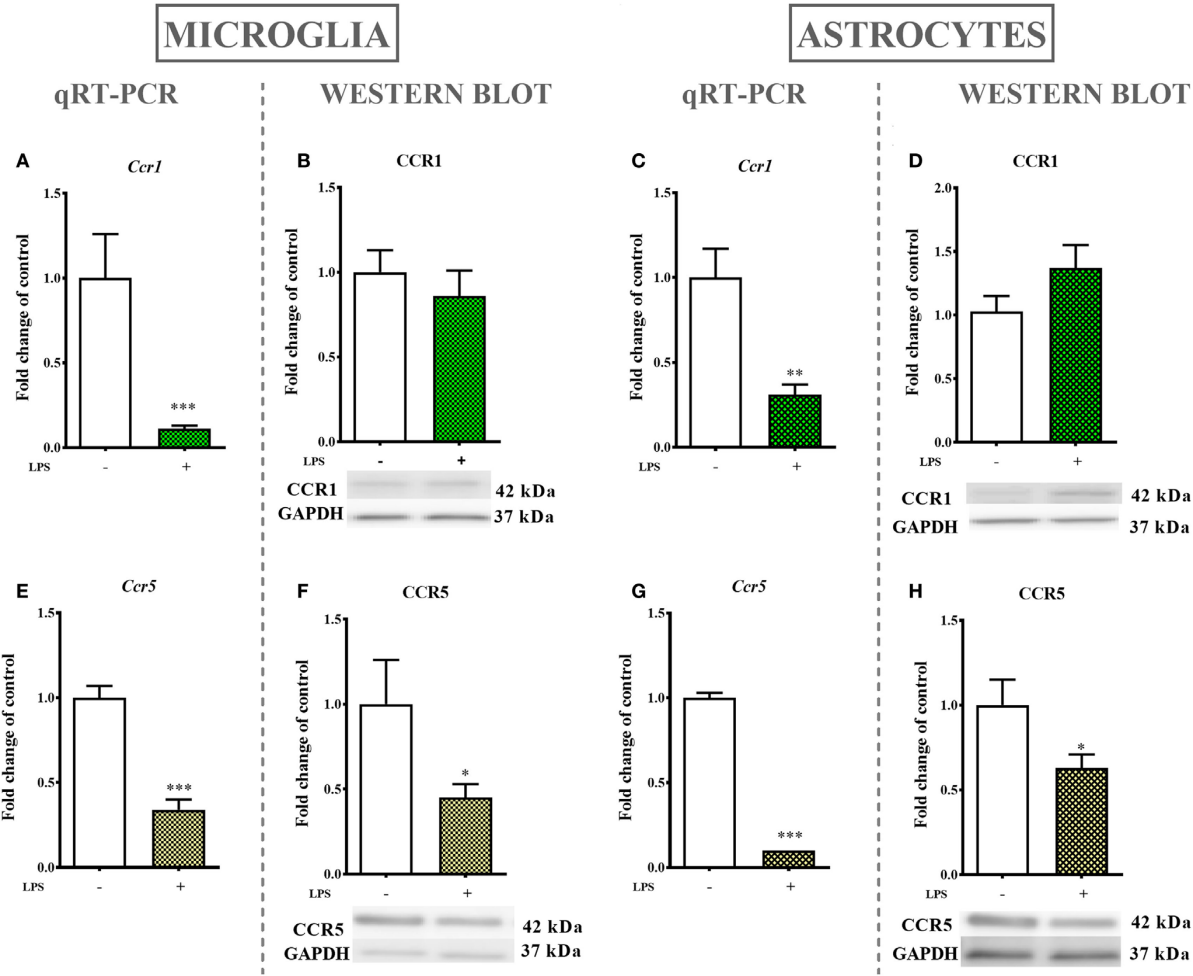
The effects of lipopolysaccharide (LPS; 100 ng/ml) stimulation on the mRNA and protein levels of CCR1 and CCR5 in primary microglial and astroglial cultures. The mRNA expression of *Ccr1* **(A,C)** and *Ccr5* **(E,G)** was measured by quantitative reverse transcriptase real-time PCR (qRT-PCR) in the vehicle(−)- and LPS(+)-treated microglia **(A,E)** and astroglia **(C,G)** at 24 h following LPS stimulation. The protein levels of CCR1 **(B,D)** and CCR5 **(F,H)** were evaluated by western blotting in the vehicle(−)- and LPS(+)-treated microglia **(B,F)** and astroglia **(D,H)** at 24 h following LPS stimulation. The data are presented as the means ± the SEM (6–12 samples per group). The results were evaluated using Student’s *t*-test; *(*P* < 0.05), **(*P* < 0.01), and ***(*P* < 0.001) indicate significant differences compared with the vehicle(−)-treated cells.

The LPS (100 ng/ml) stimulation of microglial cultures for 24 h produced a decrease in *Ccr1* (90%, Figure 6A) and *Ccr5* (65%, Figure 6E) mRNA levels as determined by qRT-PCR. Western blot protein analysis indicated that LPS stimulation for 24 h decreased the protein level of CCR5 (55%, Figure 6F) while having no influence on CCR1 expression (Figure 6B).

#### Primary Astroglial Cultures

mRNA levels in astroglial cultures stimulated by LPS (100 ng/ml) for 24 h were evaluated by qRT-PCR and showed a decrease in *Ccr1* (70%, Figure 6C) and *Ccr5* (90%, Figure 6G) expression. Western blot protein analysis after 24 h of stimulation with LPS indicated a decrease in the CCR5 (37%, Figure 6H) protein level but had no parallel influence on the CCR1 (Figure 6D) protein level.

### The Effect of Single Intrathecal (*i.t*.) Administration of CCL3 or CCL9 nAb on Nociceptive Transmission Measured on Day 7 Following STZ Injection in Mice With STZ-Induced Diabetic Neuropathy

**Figure 7:**
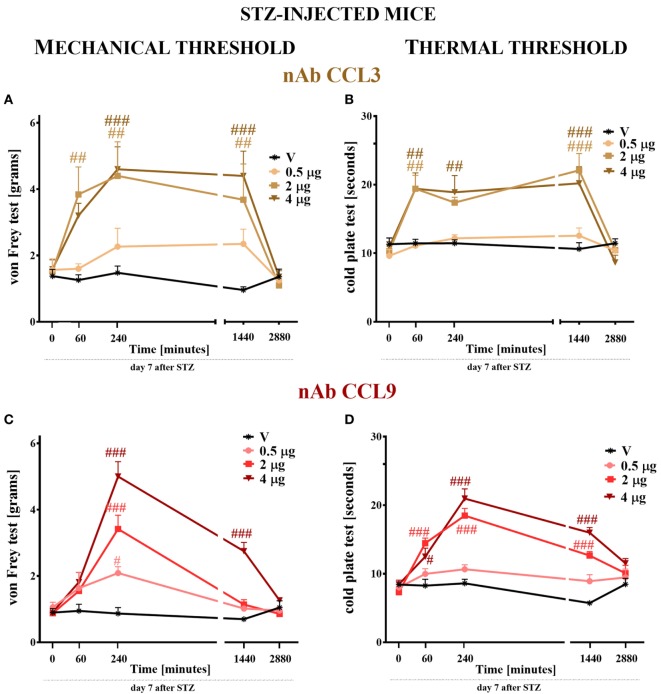
The effects of single intrathecal (*i.t*.) CCL3 or CCL9 neutralizing antibody (nAb) administration on the mechanical and thermal nociceptive thresholds in streptozotocin (STZ; 200 mg/kg; *i.p*.)-induced diabetic neuropathic mice at day 7 following STZ injection. The effects of single *i.t*. vehicle, nAb CCL3 **(A,B)** or nAb CCL9 **(C,D)** administration (0.5, 2, and 4 μg/5 μl) on the mechanical nociceptive threshold [von Frey test; **(A,C)**] and thermal nociceptive threshold [cold plate test; **(B,D)**] were measured at 60, 240, 1,440, and 2,880 min following administration at day 7 following STZ injection. The data are presented as the means ± the SEM (5–7 mice per group). The results were evaluated using two-way analysis of variance followed by Bonferroni’s test for multiple comparisons; ^#^(*P* < 0.05), ^##^(*P* < 0.01), and ^###^(*P* < 0.001) indicate significant differences compared with the vehicle-treated STZ-injected diabetic neuropathic mice.

CCL3 and CCL9 nAbs were singly administered *i.t*. on day 7 after STZ injection in the following concentrations: 0.5, 2, and 4 μg/5 μl. Additionally, control STZ-induced diabetic neuropathic mice received vehicle (V; water for injection) administration. Reactions to mechanical and thermal stimuli were assessed by von Frey (Figures 7A,C) and cold plate (Figures 7B,D) tests, respectively.

#### The Effect of CCL3 or CCL9 nAb on Mechanical Nociceptive Threshold Measured by the von Frey Test

The pain perception threshold to mechanical stimuli was elevated after the two highest doses (2 and 4 µg) of CCL3 nAb administration (Figure 7A). This effect was observed from 60 (in the case of 2 µg) or 240 (in the case of 4 µg) to 1,440 min following administration. The lower dose of this antibody did not influence the disturbance of nociceptive transmission (Figure 7A). In the case of single CCL9 nAb injection, the response to mechanical stimuli was not observed after 60 min following nAb administration; however, after 240 min each tested dose raised the threshold for reaction to mechanical stimuli in a dose-dependent manner (Figure 7C). This effect was still observed after 1,440 min, but only for the highest dose (4 µg). After 2,880 min, the pain-relieving properties of CCL3 and CCL9 nAbs were abolished (Figures 7A,C). The vehicle injection did not change the reaction to mechanical stimuli in STZ-diabetic mice (Figures 7A,C).

#### The Effect of CCL3 or CCL9 nAb on Thermal Nociceptive Threshold Measured by the Cold Plate Test

Single *i.t*. administration of 2 or 4 µg of CCL3 nAb prolonged the latency to react to thermal stimuli in a similar manner at time points up to 1,440 min following administration (Figure 6B), while the lower dose had no influence on the disturbance of nociceptive transmission. The two highest doses (2 and 4 µg) of CCL9 nAb extended the latency to react to thermal stimuli at time points up to 1,440 min. The pain-relieving effect of the 2 µg dose was stronger than that of the 4 µg dose after 60 min; however, after 240 min, the highest dose (4 µg) provides the strongest effect, and the same was observed at 1,440 min (Figure 7D). The lowest dose did not influence thermal threshold at any investigated time point (Figure 7D). Additionally, the vehicle injection did not influence thermal threshold in STZ-induced diabetic mice (Figures 7B,D).

### The Effect of a Single Intrathecal (*i.t*.) Administration of CCL3 or CCL9 nAb on Pain-Related Behavior and the Effectiveness of Morphine (M) Measured on Day 7 in Mice With STZ-Induced Diabetic Neuropathy

**Figure 8:**
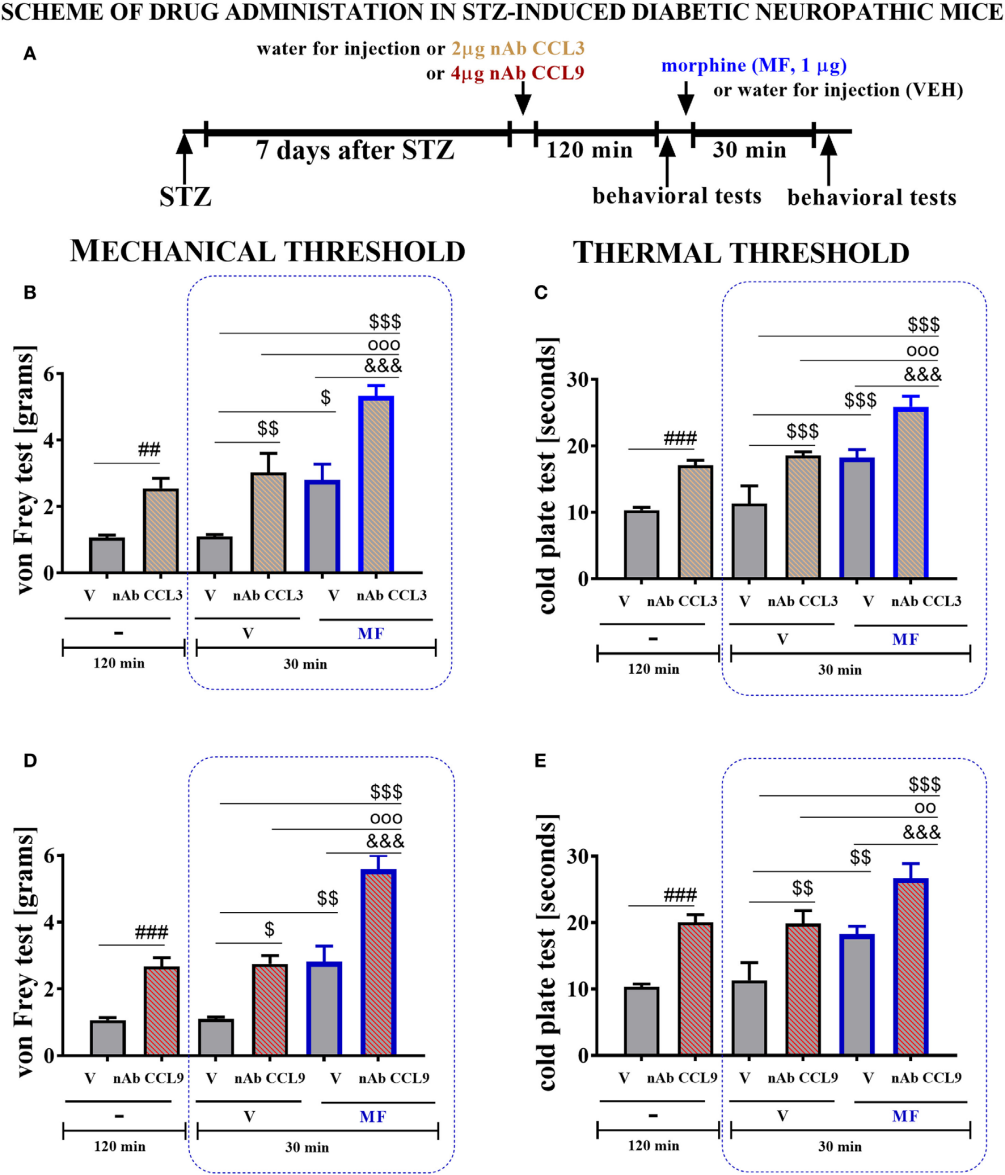
The effects of single intrathecal (*i.t*.) CCL3 or CCL9 neutralizing antibody (nAb) administration on the mechanical nociceptive threshold, the thermal nociceptive threshold and the effectiveness of morphine (M) in streptozotocin (STZ; 200 mg/kg; *i.p*.)-induced diabetic neuropathic mice at day 7 following STZ injection. Part A presents a scheme of the experiment **(A)**. The effects of single *i.t*. vehicle, nAb CCL3 [2 μg/5 μl; **(B,C)**] or nAb CCL9 [4 μg/5 μl; **(D,E)**] administration on the mechanical nociceptive threshold [von Frey test; **(B,D)**] and thermal nociceptive threshold [cold plate test; **(C,E)**] were measured 120 min following administration on day 7 following STZ injection. Morphine (1 μg/5 μl) was administered as a single *i.t*. injection 135 min after the antibody administration and the completion of the behavioral tests; the tests were repeated 30 min following the morphine administration **(B–E)**. The data are presented as the means ± the SEM (4–10 mice per group). The results were evaluated using one-way analysis of variance followed by Bonferroni’s test for multiple comparisons; ^#^(*P* < 0.05), ^##^(*P* < 0.01), and ^###^(*P* < 0.001) indicate significant differences compared with the vehicle-treated STZ-injected diabetic neuropathic mice; ^$^(*P* < 0.05), ^$$^(*P* < 0.01), and ^$$$^(*P* < 0.001) indicate significant differences compared with the vehicle (water for injection) + vehicle-treated STZ-induced diabetic neuropathic mice; ^○○^(*P* < 0.01) and ^○○○^(*P* < 0.001) indicate significant differences compared with the neutralizing antibody + vehicle-treated STZ-induced diabetic neuropathic mice. ^&&&^(*P* < 0.001) indicates significant differences compared with the vehicle + morphine-treated STZ-induced diabetic neuropathic mice.

Seven days following STZ administration, a single *i.t*. injection of vehicle (water for injection), CCL3 nAb (2 µg/5 μl), or CCL9 nAb (4 µg/5 μl) was given to STZ-induced diabetic neuropathic mice. The influence of both nAbs on the mechanical and thermal thresholds was measured by von Frey (Figures 8B,D) and cold plate (Figures 8C,E) tests, respectively, 120 min following administration (Figures 8B–E). Then, 135 min after nAb injection, a single *i.t*. administration of vehicle (water for injection) or morphine (1 µg/5 μl) was made. The von Frey (Figures 8B,D) and cold plate (Figures 8C,E) tests were performed 30 min after administration (scheme in Figure 8A).

Single *i.t*. injection of CCL3 nAb increased the nociceptive thresholds for mechanical (2.5 ± 0.3 vs. 1.1 ± 0.1 g; Figure 7B) and thermal (17.1 ± 0.8 vs. 10.3 ± 0.3 s; Figure 8C) stimuli as measured 7 days after STZ injection. Additionally, a single CCL3 nAb injection enhanced the analgesic properties of morphine against mechanical (5.3 ± 0.3g; Figure 8B) and thermal (25.9 ± 1.6s; Figure 8C) stimuli in comparison to CCL3 nAb-treated + V-injected animals (3 ± 0.6 g and 18.6 ± 0.5 s, respectively).

Single *i.t*. injection of CCL9 nAb increased the nociceptive thresholds for mechanical (2.7 ± 0.3 vs. 1.1 ± 0.1 g; Figure 8D) and thermal (20.1.1 ± 0.8 vs. 10.3 ± 0.4 s; Figure 8E) stimuli as detected 7 days after STZ injection. Additionally, single CCL9 nAb administration improved the effectiveness of morphine in the von Frey (5.6 ± 0.4 g; Figure 8D) and cold plate (26.7 ± 2.2 s; Figure 8E) tests in comparison to CCL9 nAb-treated + V-injected animals (2.8 ± 0.3 g and 19.9 ± 2 s, respectively).

Morphine administration effectively raised the pain perception threshold to both mechanical (Figures 8B,D) and thermal (Figures 8C,E) stimuli.

### The Effect of a Single Intrathecal (*i.t*.) J113863 Administration on Mechanical and Thermal Thresholds Measured on Day 7 Following STZ Administration in Mice With STZ-Induced Diabetic Neuropathy

**Figure 9:**
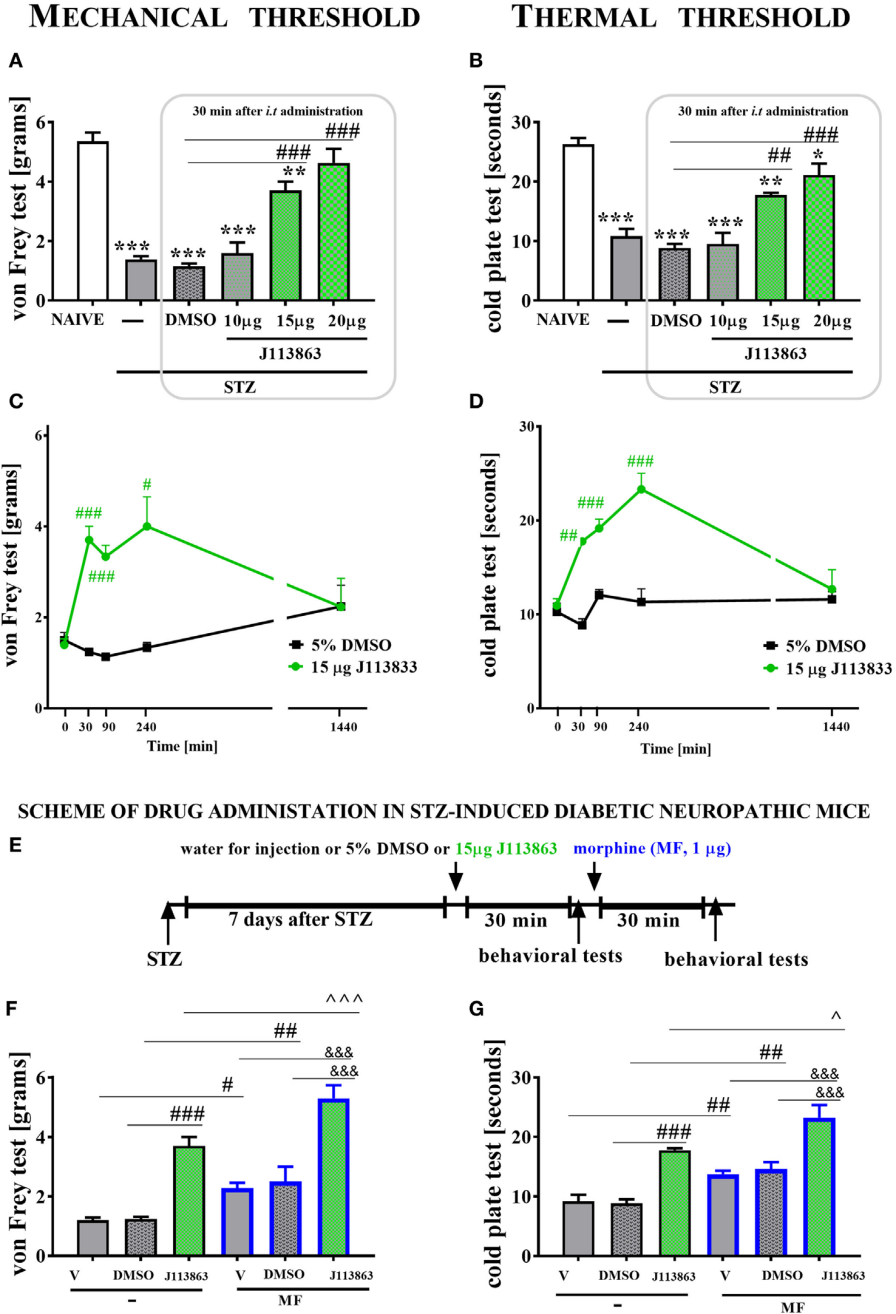
The effects of single intrathecal (*i.t*.) administration of a CCR1 antagonist (J113863) on the mechanical nociceptive threshold, the thermal nociceptive threshold and the effectiveness of morphine (M) in streptozotocin (STZ; 200 mg/kg; *i.p*.)-induced diabetic neuropathic mice at day 7 following STZ injection. The effects of single *i.t*. vehicle (5% DMSO) and J113863 [10, 15, and 20 μg/5 μl; **(A,B)**] administration on the mechanical threshold [von Frey test; **(A)**] and thermal threshold [cold plate test; **(B)**] were measured 30 min following administration on day 7 following STZ injection. The time-dependent changes of a 15 µg dose of J113863 was determined by the von Frey test **(C)** and the cold plate test **(D)** performed at 30, 90, 240, and 1,440 min following administration on day 7 after STZ injection. Scheme of combined administration of J113863 and morphine **(E)**. Morphine (1 μg/5 μl) was administered as a single *i.t*. injection 35 min after the J113863 (15 μg/5 μl) administration and the completion of the behavioral tests, and then the tests were repeated 30 min following the morphine administration **(F,G)**. The data are presented as the means ± SEM (4–14 mice per group). The results were evaluated using one-way analysis of variance followed by Bonferroni’s test for multiple comparisons; *(*P* < 0.05), **(*P* < 0.01), and ***(*P* < 0.001) indicate significant differences compared with the naïve animals; ^#^(*P* < 0.05), ^##^(*P* < 0.01), and ^###^(*P* < 0.001) indicate significant differences compared with the vehicle (5% DMSO or water for injections)-treated STZ-injected diabetic neuropathic mice; ^(*P* < 0.05) and ^^^(*P* < 0.001) indicate significant differences compared with the J113863-injected STZ-induced diabetic neuropathic mice; ^&&&^(*P* < 0.001) indicates significant differences compared with the vehicle + morphine-treated STZ-induced diabetic neuropathic mice.

Single *i.t*. administration of J113863 (a CCR1 antagonist) was performed in STZ-induced diabetic neuropathic mice at concentrations of 10, 15, and 20 µg/5 μl on day 7 after STZ injection. The control STZ-induced diabetic neuropathic mice received the vehicle (5% DMSO). The mechanical and thermal thresholds were assessed by the von Frey (Figures 9A,C) and cold plate (Figures 9B,D) tests, respectively. Additionally, for the 15 µg dose of J113863, we observed time-dependent changes in pain relief.

Streptozotocin-induced diabetic neuropathic mice developed hypersensitivity to mechanical (1.4 ± 0.1 g) and thermal (10.8 ± 1.2 s) stimuli in comparison to naïve animals (5.4 ± 0.3 g and 26.3 ± 1 s, respectively) as measured on day 7 following STZ administration (Figures 9A,B). A single injection of 10 µg of J113863 or 5% DMSO did not affect the decreased threshold of response to mechanical and thermal stimuli 30 min following administration (Figures 9A,B). In contrast, the two other doses (15 and 20 µg) of J113863 increased the response threshold to mechanical (3.7 ± 0.3 and 4.6 ± 0.5 g) and thermal (17.8 ± 0.5 and 21.1 ± 2 s) stimuli in both tests (Figures 9A,B) as measured 30 min after administration.

A single administration of 15 µg of J113863 increased the response threshold to mechanical stimuli at 30, 90, and 240 min following administration; however, the effect observed after 90 min was weaker than the ones obtained after 30 and 240 min (Figure 9C). Pain-relieving properties were no longer observed at 1,440 min (Figure 9C). The response latency to thermal stimuli was extended at 30, 90, and 240 min following administration of 15 µg of J113863. Furthermore, the analgesic effect increased up to 240 min after administration but was no longer observed after 1,440 min. A single administration of 5% DMSO in STZ-diabetic mice did not influence the mechanical or thermal threshold at any investigated time point (Figures 9C,D).

### The Effect of a Single Intrathecal *(i.t.)* J113863 Administration on the Effectiveness of Morphine Measured on Day 7 Following STZ Administration in Mice With STZ-Induced Diabetic Neuropathy

Seven days following STZ administration, a single *i.t*. injection of water for injection (V), 5% DMSO or 15 µg of J113863 was given to STZ-induced diabetic neuropathic mice, and 30 min following administration, the von Frey and cold plate tests were performed. Then, each group received a single *i.t*. injection of morphine (M; 1 µg/5 μl), and behavioral tests were performed after 30 min following morphine and 60 min following 15 µg J113863 injection (scheme in Figure 9E).

Single *i.t*. administration of 15 µg of J113863 increased the nociceptive thresholds for mechanical and thermal stimuli as assessed by the von Frey (Figure 9F) and cold plate (Figure 9G) tests, respectively, 30 min after administration. Additionally, neither water for injection nor 5% DMSO affected nociceptive transmission (Figures 9F,G).

Single *i.t*. administration of 15 µg of J113863 improved the effectiveness of morphine (1 µg; single *i.t*. injection). The elevation of the mechanical threshold after morphine treatment was more potent in J113863-injected animals (5.3 ± 0.4 g) than in V-treated (2.1 ± 0.2 g) or DMSO-injected (2.5 ± 0.5 g) animals (Figure 9F). Similarly, the reaction to thermal stimuli was delayed in morphine-treated J113863-injected mice (23.2 ± 2.1 s) compared with morphine-treated V-injected (13.8 ± 0.9 s) and morphine-treated DMSO-injected (14.6 ± 1.1 s) mice (Figure 9G). Additionally, morphine injection increased the reaction thresholds to mechanical and thermal stimuli in V-injected and DMSO-injected animals (Figures 9F,G).

### The Effect of Single Intrathecal (*i.t*.) DAPTA Administration on Mechanical and Thermal Thresholds Measured in STZ-Induced Diabetic Neuropathic Mice on Day 7 Following STZ Injection

**Figure 10:**
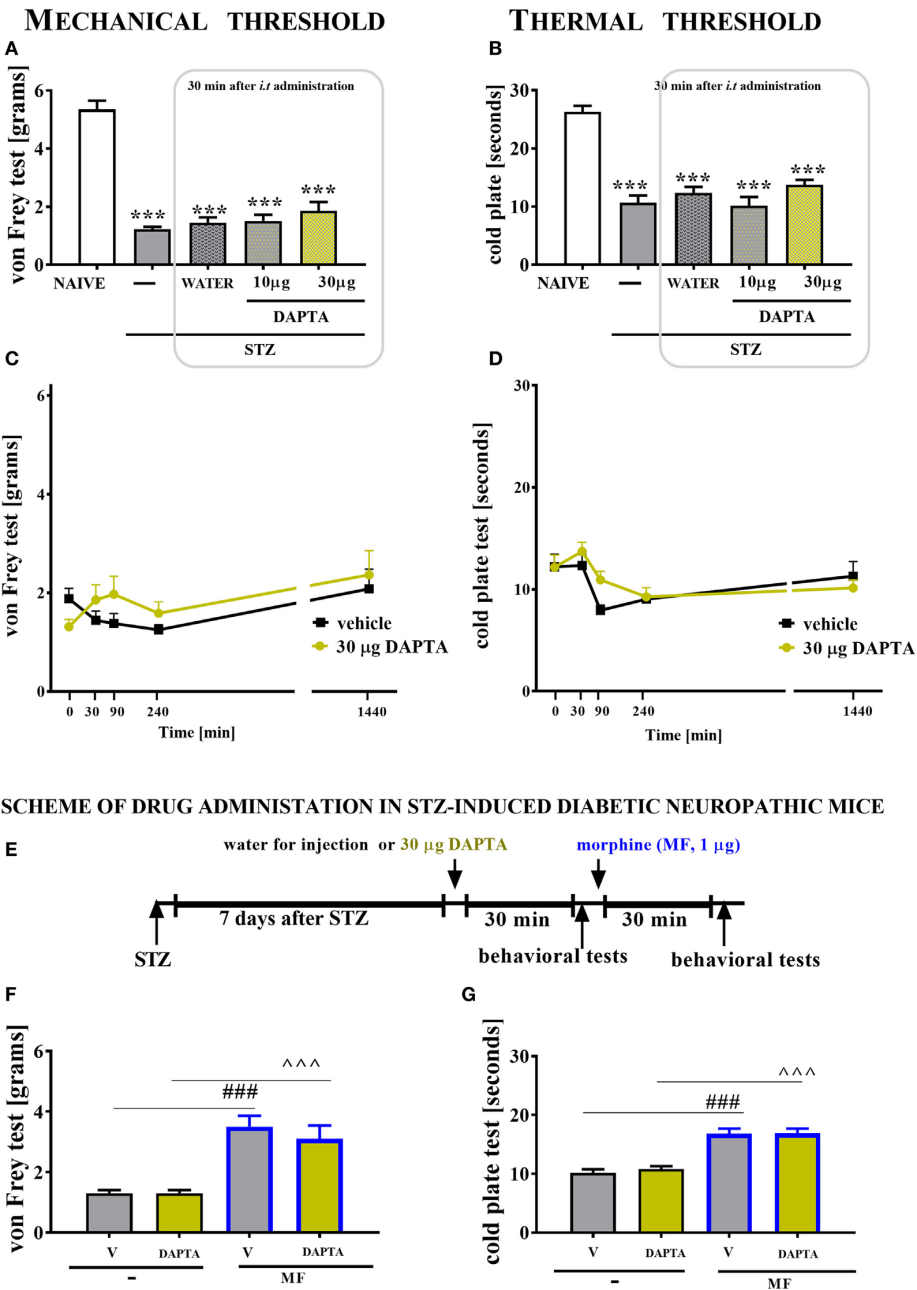
The effects of single intrathecal (*i.t*.) CCR5 antagonist (DAPTA) administration on the mechanical nociceptive threshold and thermal nociceptive threshold and the effectiveness of morphine (M) in streptozotocin (STZ; 200 mg/kg; i.p.)-induced diabetic neuropathic mice at day 7 following STZ injection. The effects of single *i.t*. vehicle (water for injection) and DAPTA [10 and 30 μg/5 μl; **(A,B)**] administration on the mechanical nociceptive threshold [von Frey test; **(A)**] and thermal nociceptive threshold [cold plate test; **(B)**] were measured 30 min following administration at day 7 following STZ injection. The time-dependent changes of a 30-µg dose of DAPTA was determined by the von Frey test **(C)** and the cold plate test **(D)** at 30, 90, 240, and 1,440 min following administration on day 7 following STZ injection. Scheme of combined administration of DAPTA and morphine **(E)**. Morphine (1 μg/5 μl) was administered as a single *i.t*. injection 35 min after the DAPTA (30 μg/5 μl) administration and the completion of the behavioral tests, and then the tests were repeated 30 min following the morphine administration **(F,G)**. The data are presented as the means ± SEM (5–9 mice per group). The results were evaluated using one-way analysis of variance followed by Bonferroni’s test for multiple comparisons; ***(*P* < 0.001) indicates significant differences compared with the naïve animals; ^^^(*P* < 0.001) indicate significant differences compared with the J113863-injected STZ-induced diabetic neuropathic mice.

Single *i.t*. administration of DAPTA (a CCR5 antagonist) was performed in STZ-induced diabetic neuropathic mice at concentrations of 10 and 30 µg/5 μl on day 7 after STZ injection. The control STZ-induced diabetic neuropathic mice received vehicle (water for injection) administration. The mechanical and thermal thresholds were assessed by the von Frey (Figures 10A,C) and cold plate (Figures 10B,D) tests, respectively.

Seven days following STZ administration, the reaction thresholds to mechanical (1.23 ± 0.1 g) and thermal (10.7 ± 1.2 s) stimuli was lower than those of naïve animals (5.3 ± 0.3 g and 26.3 ± 1 s, respectively; Figures 10A,B). A single injection of DAPTA did not influence the pain perception threshold in the von Frey test or the cold plate test, regardless of the investigated dose (Figures 10A–D, respectively). The reaction to mechanical and thermal stimuli was unchanged in morphine-treated DAPTA-injected mice compared with morphine-treated V-injected mice (Figures 10F,G). However, morphine injection increased the reaction thresholds to mechanical and thermal stimuli in V-injected animals (Figures 10F,G).

## Discussion

Our studies have indicated that single intrathecal administration of any MIP-1 member evokes strong pain-related behavior in naïve mice. Additionally, we have shown that in STZ-induced diabetes, the pain perception threshold to mechanical and thermal stimuli is lowered, which correlates with microglial activation and an increase in the expression of two MIP-1 family members (CCL3 and CCL9). Furthermore, data obtained from primary glial cell cultures suggest that all mouse MIP-1 members can originate from microglia and/or astroglial cells, and their receptors are present on those cells as well. The immunohistochemical analysis provided clear evidence that spinal CCL3 and CCL9, as well as CCR1 and CCR5, show coexpression with a neuronal marker, NeuN. However, it was hard to confirm the expression of both investigated ligands on microglial cells, as suggested by previously performed *in vitro* study. Nevertheless, apart from existing CCL3- and CCL9-positive only cells, there were also some co-localized with microglia marker, IBA1 (in particular CCL3). There was no clear co-localization observed with astroglia marker, GFAP. An additional immensely important observation concomitant with the spinal elevation of CCL3 and CCL9 expression was provided by the results of pharmacological studies. We would like to point out that the administration of nAbs for CCL3 and CCL9 dose-dependently showed analgesic effects. As we believe, it would be hard to obtain such pharmacological effects if the level of these endogenous chemokines has not been increased after STZ administration. Furthermore, antagonizing the CCR1 by single intrathecal administration of J113863 as well as by CCL3 or CCL9 neutralizing antibody in diabetic mice model leads to relief of pain-related behavior and augmentation of the effectiveness of morphine.

Chemokines are small molecules with a well-established homeostatic function based on attracting target cells to the place of their secretion. However, current studies show that exogenous administration of different chemokines, such as CCL1, CCL3, CXCL1, CXCL5, CXCL9, CXCL12, or XCL1, in naïve animals decreases the nociceptive threshold, which points to their role in pain development (9, 10, 19, 44, 45). Our results are part of this trend since we have indicated that single intrathecal administration not only of CCL3 and CCL9 but also of CCL4 to naïve mice induces the development of long-lasting hypersensitivity to mechanical and thermal stimuli, which is observed up to 24 h later. Therefore, we postulate that this immediate and long-lasting effect seems to be related to the presence of receptors for these chemokines mainly on neurons, which was also clearly shown and confirmed on immunofluorescent staining.

Previous studies have suggested that CCL3, CCL4, and CCL9 are expressed in macrophages and/or microglia (46–48), so in cells very important for diabetic pain development (6). The current study has shown that in the lumbar spinal cord of STZ-induced diabetic neuropathic mice, a marker of microglia activation is upregulated in correlation with increased protein levels of CCL3 and CCL9 as measured on day 7, suggesting that these chemokines are very important in the development of diabetic neuropathy and that their source are microglial cells. There were no changes in the CCL4 level, which is consistent with the findings of Saika et al. (16) in a partial sciatic nerve ligation model. During activation, microglial cells release a broad spectrum of spinal neuro-immunological factors, including chemokines (6, 8, 10, 19, 21, 41). Interestingly, it has been shown in many papers that observed spinal changes under neuropathic pain correlate well with the LPS-stimulated *in vitro* glial cells (8, 10, 21–23, 40, 41, 49). Primary microglial cell cultures have confirmed the presence of mRNA for each of the MIP-1 family chemokines. Additionally, the protein level of CCL3 was elevated after LPS stimulation, an observation that is supported by other studies (46). The protein analysis of CCL4, however, showed that its level was decreased after LPS treatment. In the case of CCL9, there were no significant changes in its mRNA level, and more importantly, we were not able to detect this protein in microglial culture. In 2010, Ravindran et al. showed that after stimulation of the mouse microglial cell line BV2, the mRNA level of CCL9 was increased. However, BV2 cell lines are programmed to proliferate and, therefore, their gene expression patterns may differ from those of primary microglial cells (50). The observed lack of spinal activation of GFAP-positive cells in the early phase of diabetic pain development had already been established (10, 51). However, our primary astroglial cell cultures have shown that after LPS stimulation, there was an increase in transcription and translation of CCL3 and CCL9. The lack of changes in the GFAP protein level cannot be unambiguously connected to the absence of astrocyte participation in the release of this chemokine under diabetes, especially since it was indicated that changes in the level of GFAP are not related to the number of astrocytes (52).

Immunohistochemical staining can dispel the doubts arising from the difference between the data obtained from primary glial cell cultures and those from the lumbar spinal cord of STZ-induced diabetic neuropathic mice. Our results clearly show that CCL3 and CCL9 co-localize with a neuronal marker, which can explain the spinal increase of CCL9 protein, pointing to neurons as main source of this chemokine. In the case of CCL3, the involvement of neurons, not just microglia, seems to be highly important. Of course, as suggested by mentioned above *in vitro* data (partially confirmed by immunohistochemistry), one cannot exclude the microglial cells as an alternative origin of both investigated chemokines, in particular CCL3. This could have been hard to confirm, as even STZ-induced expression of microglial cells is relatively very low in comparison to, i.e., effect observed in neurodegeneration or other inflammatory processes (8, 21, 53).

In general, a distinctive feature of chemokines is their ability to interact with more than one G-protein-coupled chemokine receptor and *vice versa*. An analogous situation occurs in the case of MIP-1 family members, which interact with CCR1, CCR5, or both (15). In the central nervous system, those receptors are expressed on neurons (54, 55), microglia (54, 56), and astrocytes (54, 57). The results obtained from the immunohistochemical staining confirm the spinal co-localization of NeuN with CCR1 and CCR5 of STZ-induced diabetic mice. The spinal neuronal localization of these receptors confirmed their important role in nociceptive transmission and explained why we observed immediate pronociceptive effects of CCL3, CCL4 and CCL9 after their intrathecal administration. Furthermore, primary glial cell cultures demonstrated that both of these receptors are localized on microglia and astroglia, which is consistent with the findings of others (57–59) and was partially confirmed by our immunohistochemical study. Furthermore, we found that, during the development of neuropathy in our STZ-induced diabetes model, the protein levels of CCR1 and CCR5 are not changed.

Interestingly, LPS stimulation of primary glial cell cultures results in a decrease of *Ccr1* and *Ccr5* mRNA expression accompanied by downregulation of the CCR5 protein level but no change in the CCR1 protein level. Those findings are different from those of Boddeke et al., who have shown that 12 h of LPS (100 ng/ml) treatment upregulates the mRNA levels of *Ccr1* and *Ccr5* in rat microglial cultures (58). Others, however, demonstrated that the expression of *Ccr5* mRNA was not changed when BV2 cells were used (60). Those discrepancies emphasize the complexity of intracellular processes in which G-protein-coupled receptors are involved; these processes should be explored more deeply.

The pain-relieving properties of different nAbs are well studied since there are used to delay paclitaxel-induced neuropathy (61) and bone cancer pain (18). Our results demonstrated that nAbs for CCL3 and CCL9 not only raises the nociceptive threshold but also enhances the effectiveness of morphine. Therefore, defining the role of this chemokines seems to be highly important, especially because autoantibodies against CCL3 have been postulated to be a biomarker of type 1 diabetes development in humans (14).

Treatment of diabetic neuropathic pain is difficult and commonly ineffective. Therefore, we focused our attention on antagonizing MIP-1 family receptors, CCR1 and CCR5, by J113863 and DAPTA administration, respectively. The results of our study in an STZ-induced diabetic neuropathy model show that single intrathecal administration of the CCR1 antagonist J113863 dose-dependently diminished pain-related behavior in STZ-injected mice. Similarly, it has been shown that blockade of CCR1 dose-dependently reduces pain perception of mechanical and thermal stimuli in a complete Freund’s adjuvant inflammatory pain mouse model (62). Surprisingly, the CCR5 antagonist DAPTA has no analgesic properties in STZ model. Apparently, the data obtained in diabetic and non-diabetic neuropathic pain model seem to be different. For example, Saika et al. (16) have shown that DAPTA after partial sciatic nerve ligation attenuates hypersensitivity in mice. Additionally, also our studies have indicated that maraviroc, another CCR5 antagonist, can attenuate the development of neuropathic pain symptoms in rats after sciatic nerve ligation (21, 22). The observed differences between the analgesic effects of CCR1 and CCR5 antagonists in STZ-induced neuropathy are highly interesting. The observed lack of changes in the level of CCR1 in our model was initially baffling. However, we can hypothesize that exposure of the G-protein-coupled receptors CCR1 to agonists (CCL3 and CCL9) leads to a rapid decrease in the number of cell-surface binding sites. Therefore, we did not observe an increased level of CCR1, which as suggested by our pharmacological experiments, is strongly involved in nociceptive transmission and plays a crucial role in diabetic neuropathy. This fact underlines the importance of better understanding G-coupled receptor signaling, which is known to be important in the context of mechanisms of action of various factors. The activation of the G-coupled receptors leads to cellular signal transduction through many intracellular pathways, e.g., MAPKs, NF-κB, STATs, PI3K (6, 23, 40, 41, 63–66). Recent studies have suggested that one of the most important intracellular pathways is the PI3K-pathway, initiated by many cytokines as well as insulin. Dysregulation of aforementioned pathway is implicated by some authors in non-diabetic (67) and diabetic (68) neuropathic pain models. The involvement of different intracellular pathways in CCR1 function under diabetes undoubtedly should be examined.

Morphine is an opioid receptor agonist used in case of moderate or severe pain states (69); however, its efficacy is lost in long-lasting pain conditions including diabetic neuropathy (70). Recent studies have shown that chronic morphine treatment leads to loss of its effectiveness in neuropathic mice, which is correlated with the degree of severity of microglial cell activation (63). Interestingly, combined administration with microglial inhibitors prolongs the analgesic effect of morphine (6, 63). Base on this phenomenon, current studies are focused on searching for the substances that influence activated microglia in parallel with enhancing the effectiveness of morphine. It has been reported that after sciatic nerve ligation, a CCR5 antagonist simultaneously prevented pain-related behavior development and glia activation while also enhancing the efficacy of morphine (22); however, in diabetic neuropathy was not as effective. In contrast, the results of the current study indicate that, in diabetic neuropathic pain, the CCR1 antagonist J113863 not only relieves pain-related behavior but also improves the analgesic properties of morphine, emphasizing once again the important role of this receptor in our diabetic neuropathic pain model. Based on current literature data we can only speculate, that the enhancement of morphine analgesia evoked by J113863 is associated with the fact that in neurons CCR1 is coexpressed with the mu opioid receptor—as it was already shown that the activation of CCR1 leads to internalization of mu opioid receptor which markedly inhibits its function (71). However, this needs future investigation in other diabetic pain models.

## Conclusion

In summary, our behavioral and biochemical studies highlight a crucial role of two MIP-1 family chemokines in neuropathic pain accompanying diabetes and point to a key role of CCR1. We give evidence that CCL3 and CCL9, based on their spinal upregulation and strong pronociceptive properties, might be important factors involved in the development of diabetic neuropathy. It was postulated that autoantibodies against CCL3 are biomarkers of type 1 diabetes development (14), our results suggest that CCL9 may share this function. Additionally, their direct neutralization not only attenuates neuropathic pain symptoms but also enhance the effectiveness of morphine, which may be crucial for future progress in therapy. Moreover, our results suggest the pharmacological blockade of CCR1 in conjunction with morphine administration as a novel therapeutic approach for diabetic neuropathy.

## Ethics Statement

The number of animals was limited to the minimum necessary. The experiments were carried out according to IASP recommendations (24), NIH Guide for Care and Use of Laboratory Animals and approved by the 2nd Local Ethical Committee on Animal Testing in Institute of Pharmacology, Polish Academy of Sciences (12 Smetna Str., 31-343 Krakow, Poland; permission number: 1277/2015 and 75/2017).

## Author Contributions

ER, MZ, AP, GK, and JM made the experiments. ER, MZ, and JM planned the study ER, MZ, AP, GK, IN, and JM analyzed and interpreted the results, drafted the manuscript, and accepted the finalized version.

## Conflict of Interest Statement

The authors declare that the research was conducted in the absence of any commercial or financial relationships that could be construed as a potential conflict of interest.

## References

[1] Javed S Alam U Malik RA. Treating diabetic neuropathy: present strategies and emerging solutions. Rev Diabet Stud (2015) 12:63–83. 10.1900/RDS.2015.12.63 26676662 PMC5397984

[2] Lotfy M Adeghate J Kalasz H Singh J Adeghate E. Chronic complications of diabetes mellitus: a mini review. Curr Diabetes Rev (2017) 13:3–10. 10.2174/1573399812666151016101622 26472574

[3] Chen YW Chiu CC Hsieh PL Hung CH Wang JJ. Treadmill training combined with insulin suppresses diabetic nerve pain and cytokines in rat sciatic nerve. Anesth Analg (2015) 121:239–46. 10.1213/ANE.0000000000000799 25993391

[4] Pabreja K Dua K Sharma S Padi SS Kulkarni SK. Minocycline attenuates the development of diabetic neuropathic pain: possible anti-inflammatory and anti-oxidant mechanisms. Eur J Pharmacol (2011) 661:15–21. 10.1016/j.ejphar.2011.04.014 21536024

[5] Tang W Lv Q Chen XF Zou JJ Liu ZM Shi YQ. CD8(+) T cell-mediated cytotoxicity toward Schwann cells promotes diabetic peripheral neuropathy. Cell Physiol Biochem (2013) 32:827–37. 10.1159/000354485 24080983

[6] Zychowska M Rojewska E Kreiner G Nalepa I Przewlocka B Mika J. Minocycline influences the anti-inflammatory interleukins and enhances the effectiveness of morphine under mice diabetic neuropathy. J Neuroimmunol (2013) 262:35–45. 10.1016/j.jneuroim.2013.06.005 23870534

[7] Kiguchi N Maeda T Kobayashi Y Fukazawa Y Kishioka S. Macrophage inflammatory protein-1alpha mediates the development of neuropathic pain following peripheral nerve injury through interleukin-1beta up-regulation. Pain (2010) 149:305–15. 10.1016/j.pain.2010.02.025 20223588

[8] Piotrowska A Kwiatkowski K Rojewska E Slusarczyk J Makuch W Basta-Kaim A Direct and indirect pharmacological modulation of CCL2/CCR2 pathway results in attenuation of neuropathic pain – in vivo and in vitro evidence. J Neuroimmunol (2016) 297:9–19. 10.1016/j.jneuroim.2016.04.017 27397071

[9] Zychowska M Rojewska E Pilat D Mika J. The role of some chemokines from the CXC subfamily in a mouse model of diabetic neuropathy. J Diabetes Res (2015) 2015:750182. 10.1155/2015/750182 25789329 PMC4350880

[10] Zychowska M Rojewska E Piotrowska A Kreiner G Mika J. Microglial inhibition influences XCL1/XCR1 expression and causes analgesic effects in a mouse model of diabetic neuropathy. Anesthesiology (2016) 125:573–89. 10.1097/ALN.0000000000001219 27387353

[11] Zhao J Wang H Song T Yang Y Gu K Ma P Thalidomide promotes morphine efficacy and prevents morphine-induced tolerance in rats with diabetic neuropathy. Neurochem Res (2016) 41:3171–80. 10.1007/s11064-016-2041-7 27573481

[12] Castany S Carcolé M Leánez S Pol O. The antinociceptive effects of a δ-opioid receptor agonist in mice with painful diabetic neuropathy: involvement of heme oxygenase 1. Neurosci Lett (2016) 614:49–54. 10.1016/j.neulet.2015.12.059 26762785

[13] Kou ZZ Wan FP Bai Y Li CY Hu JC Zhang GT Decreased endomorphin-2 and µ-opioid receptor in the spinal cord are associated with painful diabetic neuropathy. Front Mol Neurosci (2016) 9:80 10.3389/fnmol.2016.00080 27656127 PMC5013037

[14] Shehadeh N Pollack S Wildbaum G Zohar Y Shafat I Makhoul R Selective autoantibody production against CCL3 is associated with human type 1 diabetes mellitus and serves as a novel biomarker for its diagnosis. J Immunol (2009) 182:8104–9. 10.4049/jimmunol.0803348 19494336

[15] Maurer M von Stebut E. Macrophage inflammatory protein-1. Int J Biochem Cell Biol (2004) 36:1882–6. 10.1016/j.biocel.2003.10.019 15203102

[16] Saika F Kiguchi N Kobayashi Y Fukazawa Y Kishioka S CC-chemokine ligand 4/macrophage inflammatory protein-1β participates in the induction of neuropathic pain after peripheral nerve injury. Eur J Pain (2012) 16:1271–80. 10.1002/j.1532-2149.2012.00146.x 22528550

[17] Dawes JM Kiesewetter H Perkins JR Bennett DL McMahon SB. Chemokine expression in peripheral tissues from the monosodium iodoacetate model of chronic joint pain. Mol Pain (2013) 9:57. 10.1186/1744-8069-9-57 24206615 PMC3835139

[18] Wang ZL Du TT Zhang RG. JNK in spinal cord facilitates bone cancer pain in rats through modulation of CXCL1. J Huazhong Univ Sci Technolog Med Sci (2016) 36:88–94. 10.1007/s11596-016-1547-1 26838746

[19] Zychowska M Rojewska E Piotrowska A Kreiner G Nalepa I Mika J. Spinal CCL1/CCR8 signaling interplay as a potential therapeutic target – evidence from a mouse diabetic neuropathy model. Int Immunopharmacol (2017) 52:261–71. 10.1016/j.intimp.2017.09.021 28961489

[20] Serrano A Paré M McIntosh F Elmes SJ Martino G Jomphe C Blocking spinal CCR2 with AZ889 reversed hyperalgesia in a model of neuropathic pain. Mol Pain (2010) 6:90. 10.1186/1744-8069-6-90 21143971 PMC3009975

[21] Piotrowska A Kwiatkowski K Rojewska E Makuch W Mika J Maraviroc reduces neuropathic pain through polarization of microglia and astroglia – evidence from in vivo and in vitro studies. Neuropharmacology (2016) 108:207–19. 10.1016/j.neuropharm.2016.04.024 27117708

[22] Kwiatkowski K Piotrowska A Rojewska E Makuch W Jurga A Slusarczyk J Beneficial properties of maraviroc on neuropathic pain development and opioid effectiveness in rats. Prog Neuropsychopharmacol Biol Psychiatry (2016) 64:68–78. 10.1016/j.pnpbp.2015.07.005 26190414

[23] Kwiatkowski K Piotrowska A Rojewska E Makuch W Mika J. The RS504393 influences the level of nociceptive factors and enhances opioid analgesic potency in neuropathic rats. J Neuroimmune Pharmacol (2017) 12:402–19. 10.1007/s11481-017-9729-6 28337574 PMC5527054

[24] Zimmermann M Ethical guidelines for investigations of experimental pain in conscious animals. Pain (1983) 16:109–10. 10.1016/0304-3959(83)90201-4 6877845

[25] Dogrul A Gul H Yesilyurt O Ulas UH Yildiz O. Systemic and spinal administration of etanercept, a tumor necrosis factor alpha inhibitor, blocks tactile allodynia in diabetic mice. Acta Diabetol (2011) 48:135–42. 10.1007/s00592-010-0237-x 21104419

[26] Rebalka IA Cao AW Raleigh MJ Henriksbo BD Coleman SK Schertzer JD Statin therapy negatively impacts skeletal muscle regeneration and cutaneous wound repair in type 1 diabetic mice. Front Physiol (2017) 8:1088 10.3389/fphys.2017.01088 29311999 PMC5742241

[27] Gabra BH Sirois P. Beneficial effect of chronic treatment with the selective bradykinin B1 receptor antagonists, R-715 and R-954, in attenuating streptozotocin-diabetic thermal hyperalgesia in mice. Peptides (2003) 24:1131–9. 10.1016/j.peptides.2003.06.003 14612183

[28] Gezginci-Oktayoglu S Sacan O Yanardag R Karatug A Bolkent S. Exendin-4 improves hepatocyte injury by decreasing proliferation through blocking NGF/TrkA in diabetic mice. Peptides (2011) 32:223–31. 10.1016/j.peptides.2010.10.025 21055431

[29] Kanki H Fukuda K Okushi K Ibata I Toyama Y Shimizu H Comparison of nerve growth factor mRNA expression in cardiac and skeletal muscle in streptozotocin-induced diabetic mice. Life Sci (1999) 65(22): 2305–13. 10597885 10.1016/s0024-3205(99)00497-x

[30] Migita K Moriyama T Koguchi M Honda K Katsuragi T Takano Y Modulation of P2X receptors in dorsal root ganglion neurons of streptozotocin-induced diabetic neuropathy. Neurosci Lett (2009) 13(452):200–3. 10.1016/j.neulet.2009.01.048 19383439

[31] Murakami T Iwanaga T Ogawa Y Fujita Y Sato E Yoshitomi H Development of sensory neuropathy in streptozotocin-induced diabetic mice. Brain Behav (2013) 3:35–41. 10.1002/brb3.111 23407314 PMC3568788

[32] Ohsawa M Aasato M Hayashi SS Kamei J. RhoA/Rho kinase pathway contributes to the pathogenesis of thermal hyperalgesia in diabetic mice. Pain (2011) 152:114–22. 10.1016/j.pain.2010.10.005 20980102

[33] Valsecchi AE Franchi S Panerai AE Rossi A Sacerdote P Colleoni M. The soy isoflavone genistein reverses oxidative and inflammatory state, neuropathic pain, neurotrophic and vasculature deficits in diabetes mouse model. Eur J Pharmacol (2011) 15:694–702. 10.1016/j.ejphar.2010.10.060 21050844

[34] Lenzen S. The mechanisms of alloxan- and streptozotocin-induced diabetes. Diabetologia (2008) 51:216–26. 10.1007/s00125-007-0886-7 18087688

[35] Bishnoi M Bosgraaf CA Abooj M Zhong L Premkumar LS. Streptozotocin-induced early thermal hyperalgesia is independent of glycemic state of rats: role of transient receptor potential vanilloid 1(TRPV1) and inflammatory mediators. Mol Pain (2011) 7:52. 10.1186/1744-8069-7-52 21794120 PMC3157448

[36] Osikowicz M Skup M Mika J Makuch W Czarkowska-Bauch J Przewlocka B.Glial inhibitors influence the mRNA and protein levels of mGlu2/3, 5 and 7 receptors and potentiate the analgesic effects of their ligands in a mouse model of neuropathic pain. Pain (2009) 147:175–86. 10.1016/j.pain.2009.09.002 19782473

[37] Mika J Osikowicz M Makuch W Przewlocka B. Minocycline and pentoxifylline attenuate allodynia and hyperalgesia and potentiate the effects of morphine in rat and mouse models of neuropathic pain. Eur J Pharmacol (2007) 560:142–9. 10.1016/j.ejphar.2007.01.013 17307159

[38] Hylden JL Wilcox GL. Intrathecal morphine in mice: a new technique. Eur J Pharmacol (1980) 67:313–6. 10.1016/0014-2999(80)90515-4 6893963

[39] Zawadzka M Kaminska B. A novel mechanism of FK506-mediated neuroprotection: downregulation of cytokine expression in glial cells. Glia (2005) 49:36–51. 10.1002/glia.20092 15390105

[40] Rojewska E Popiolek-Barczyk K Jurga AM Makuch W Przewlocka B Mika J. Involvement of pro- and antinociceptive factors in minocycline analgesia in rat neuropathic pain model. J Neuroimmunol (2014) 277:57–66. 10.1016/j.jneuroim.2014.09.020 25304927

[41] Popiolek-Barczyk K Kolosowska N Piotrowska A Makuch W Rojewska E Jurga AM Parthenolide relieves pain and promotes M2 microglia/macrophage polarization in rat model of neuropathy. Neural Plast (2015) 2015:676473. 10.1155/2015/676473 26090236 PMC4452088

[42] Chomczynski P Sacchi N. Single-step method of RNA isolation by acid guanidinium thiocyanate-phenol-chloroform extraction. Anal Biochem (1987) 162:156–9. 10.1006/abio.1987.9999 2440339

[43] Chmielarz P Kuśmierczyk J Parlato R Schütz G Nalepa I Kreiner G. Inactivation of glucocorticoid receptor in noradrenergic system influences anxiety- and depressive-like behavior in mice. PLoS One (2013) 8:e72632. 10.1371/journal.pone.0072632 23977333 PMC3748181

[44] Matsushita K Tozaki-Saitoh H Kojima C Masuda T Tsuda M Inoue K Chemokine (C-C motif) receptor 5 is an important pathological regulator in the development and maintenance of neuropathic pain. Anesthesiology (2014) 120:1491–503. 10.1097/ALN.0000000000000190 24589480

[45] Reaux-Le Goazigo A Rivat C Kitabgi P Pohl M Melik Parsadaniantz S. Cellular and subcellular localization of CXCL12 and CXCR4 in rat nociceptive structures: physiological relevance. Eur J Neurosci (2012) 36:2619–31. 10.1111/j.1460-9568.2012.08179.x 22694179

[46] Kataoka A Tozaki-Saitoh H Koga Y Tsuda M Inoue K. Activation of P2X7 receptors induces CCL3 production in microglial cells through transcription factor NFAT. J Neurochem (2009) 108:115–25. 10.1111/j.1471-4159.2008.05744.x 19014371

[47] Simpson JE Newcombe J Cuzner ML Woodroofe MN. Expression of monocyte chemoattractant protein-1 and other beta-chemokines by resident glia and inflammatory cells in multiple sclerosis lesions. J Neuroimmunol (1998) 84:238–49. 10.1016/S0165-5728(97)00208-7 9628469

[48] Ravindran C Cheng YC Liang SM. CpG-ODNs induces up-regulated expression of chemokine CCL9 in mouse macrophages and microglia. Cell Immunol (2010) 260:113–8. 10.1016/j.cellimm.2009.10.001 19883904

[49] Wang YW Zhang X Chen CL Liu QZ Xu JW Qian QQ Protective effects of garcinol against neuropathic pain – evidence from in vivo and in vitro studies. Neurosci Lett (2017) 24(647):85–90. 10.1016/j.neulet.2017.03.015 28302538

[50] Prinz M Priller J. Microglia and brain macrophages in the molecular age: from origin to neuropsychiatric disease. Nat Rev Neurosci (2014) 15:300–12. 10.1038/nrn3722 24713688

[51] Liao YH Zhang GH Jia D Wang P Qian NS He F Spinal astrocytic activation contributes to mechanical allodynia in a mouse model of type 2 diabetes. Brain Res (2011) 1368:324–35. 10.1016/j.brainres.2010.10.044 20971097

[52] Coleman E Judd R Hoe L Dennis J Posner P. Effects of diabetes mellitus on astrocyte GFAP and glutamate transporters in the CNS. Glia (2004) 48:166–78. 10.1002/glia.20068 15378652

[53] Xue QS Yang C Hoffman PM Streitm WJ. Microglial response to murine leukemia virus-induced encephalopathy is a good indicator of neuronal perturbations. Brain Res (2010) 1319:131–41. 10.1016/j.brainres.2009.12.089 20059990 PMC2826545

[54] Rottman JB Ganley KP Williams K Wu L Mackay CR Ringler DJ. Cellular localization of the chemokine receptor CCR5. Correlation to cellular targets of HIV-1 infection. Am J Pathol (1997) 151:1341–51. 9358760 PMC1858074

[55] Zhang N Inan S Cowan A Sun R Wang JM Rogers TJ A proinflammatory chemokine, CCL3, sensitizes the heat- and capsaicin-gated ion channel TRPV1. Proc Natl Acad Sci U S A (2005) 102:4536–41. 10.1073/pnas.0406030102 15764707 PMC555471

[56] Eltayeb S Berg AL Lassmann H Wallström E Nilsson M Olsson T Temporal expression and cellular origin of CC chemokine receptors CCR1, CCR2 and CCR5 in the central nervous system: insight into mechanisms of MOG-induced EAE. J Neuroinflammation (2007) 4:14. 10.1186/1742-2094-4-14 17484785 PMC1884136

[57] Knerlich-Lukoschus F Chemokines and their receptors: important mediators to be aware of in neuroregenerative approaches for spinal cord injury. Neural Regen Res (2015) 10:562–4. 10.4103/1673-5374.155423 26170814 PMC4424746

[58] Boddeke EW Meigel I Frentzel S Gourmala NG Harrison JK Buttini M Cultured rat microglia express functional beta-chemokine receptors. J Neuroimmunol (1999) 98:176–84. 10.1016/S0165-5728(99)00096-X 10430051

[59] Kaul M Ma Q Medders KE Desai MK Lipton SA. HIV-1 coreceptors CCR5 and CXCR4 both mediate neuronal cell death but CCR5 paradoxically can also contribute to protection. Cell Death Differ (2007) 14:296–305. 10.1038/sj.cdd.4402006 16841089

[60] Kremlev SG Roberts RL Palmer C. Differential expression of chemokines and chemokine receptors during microglial activation and inhibition. J Neuroimmunol (2004) 149:1–9. 10.1016/j.jneuroim.2003.11.012 15020059

[61] Ochi-ishi R Nagata K Inoue T Tozaki-Saitoh H Tsuda M Inoue K. Involvement of the chemokine CCL3 and the purinoceptor P2X7 in the spinal cord in paclitaxel-induced mechanical allodynia. Mol Pain (2014) 10:53. 10.1186/1744-8069-10-53 25127716 PMC4141668

[62] Llorián-Salvador M González-Rodríguez S Lastra A Fernández-García MT Hidalgo A Menéndez L Involvement of CC chemokine receptor 1 and CCL3 in acute and chronic inflammatory pain in mice. Basic Clin Pharmacol Toxicol (2016) 119:32–40. 10.1111/bcpt.12543 26663750

[63] Mika J Wawrzczak-Bargiela A Osikowicz M Makuch W Przewlocka B. Attenuation of morphine tolerance by minocycline and pentoxifylline in naive and neuropathic mice. Brain Behav Immun (2009) 23:75–84. 10.1016/j.bbi.2008.07.005 18684397

[64] Mika J Zychowska M Popiolek-Barczyk K Rojewska E Przewlocka B Importance of glial activation in neuropathic pain. Eur J Pharmacol (2013) 15(716):106–19. 10.1016/j.ejphar.2013.01.072 23500198

[65] Popiolek-Barczyk K Mika J. Targeting the microglial signaling pathways: new insights in the modulation of neuropathic pain. Curr Med Chem (2016) 23:2908–28. 10.2174/0929867323666160607120124 27281131 PMC5427777

[66] Rojewska E Popiolek-Barczyk K Kolosowska N Piotrowska A Zychowska M Makuch W PD98059 influences immune factors and enhances opioid analgesia in model of neuropathy. PLoS One (2015) 1(10):e0138583. 10.1371/journal.pone.0138583 26426693 PMC4591269

[67] Liu W Lv Y Ren F. PI3K/Akt pathway is required for spinal central sensitization in neuropathic pain. Cell Mol Neurobiol (2017). 10.1007/s10571-017-0541-x 28849293 PMC11481961

[68] Jiang Y Mizisin AP Rearden A Jolivalt CG. Diabetes induces changes in ILK, PINCH and components of related pathways in the spinal cord of rats. Brain Res (2010) 21(1332):100–9. 10.1016/j.brainres.2010.03.067 20347724 PMC2866122

[69] Martin TJ Eisenach JC. Pharmacology of opioid and nonopioid analgesics in chronic pain states. J Pharmacol Exp Ther (2001) 299:811–7. 11714863

[70] Chen SR Pan HL. Antinociceptive effect of morphine, but not mu opioid receptor number, is attenuated in the spinal cord of diabetic rats. Anesthesiology (2003) 99:1409–14. 10.1097/00000542-200312000-00026 14639157

[71] Zhang N Rogers TJ Caterina M Oppenheim JJ. Proinflammatory chemokines, such as C-C chemokine ligand 3, desensitize mu-opioid receptors on dorsal root ganglia neurons. J Immunol (2004) 173:594–9. 10.4049/jimmunol.173.1.594 15210821

